# Characterization of novel mevalonate kinases from the tardigrade *Ramazzottius varieornatus* and the psychrophilic archaeon *Methanococcoides burtonii*

**DOI:** 10.1107/S2059798324001360

**Published:** 2024-02-27

**Authors:** Lygie Esquirol, Janet Newman, Tom Nebl, Colin Scott, Claudia Vickers, Frank Sainsbury, Thomas S. Peat

**Affiliations:** a Environment, Commonwealth Scientific and Industrial Research Organisation, GPO Box 1700, Canberra, ACT 2601, Australia; b School of Biotechnology and Biomolecular Sciences, University of New South Wales, Sydney, NSW 2052, Australia; c Manufacturing, Commonwealth Scientific and Industrial Research Organisation, GPO Box 1700, Canberra, ACT 2601, Australia; d Advanced Engineering Biology Future Science Platform, Commonwealth Scientific and Industrial Research Organisation, GPO Box 1700, Canberra, ACT 2601, Australia; e Centre for Cell Factories and Biopolymers, Griffith Institute for Drug Discovery, Griffith University, Brisbane, QLD 4111, Australia; f Synbio Future Science Platform, Commonwealth Scientific and Industrial Research Organisation, GPO Box 1700, Canberra, ACT 2601, Australia; g Queensland University of Technology, Brisbane, QLD 4000, Australia; University of Queensland, Australia

**Keywords:** mevalonate kinases, GHMP enzyme superfamily, feedback inhibition, *Ramazzottius varieornatus*, *Methanococcoides burtonii*

## Abstract

Mevalonate kinase is central to the isoprenoid biosynthesis pathway. Here, high-resolution X-ray crystal structures of two mevalonate kinases are presented: a eukaryotic protein from *Ramazzottius varieornatus* and an archaeal protein from *Methanococcoides burtonii*. Both enzymes possess the highly conserved motifs of the GHMP enzyme superfamily, with notable differences between the two enzymes in the N-terminal part of the structures. Biochemical characterization of the two enzymes revealed major differences in their sensitivity to geranyl pyrophosphate and farnesyl pyrophosphate, and in their thermal stabilities. This work adds to the understanding of the structural basis of enzyme inhibition and thermostability in mevalonate kinases.

## Abstract 2

This work reports the purification, biochemical characterization and high-resolution structures of two novel mevalonate kinases: one from the extremotolerant tardigrade *Ramazzottius varieornatus* at 2 Å resolution and one from the psychrophilic archaeon *Methanococcoides burtonii* at 2.2 Å resolution.

## 1. Introduction

The isoprenoid biosynthesis pathway is found across the archaeal, eubacterial and eukaryotic kingdoms (Lombard & Moreira, 2011 ▸). It is essential for life as it is involved in the production of cholesterol (Gabor & Fessler, 2017 ▸) and vitamins (Holstein & Hohl, 2004 ▸), as well as in the production of secondary metabolites ranging from defensive compounds such as phytoalexins (Ahuja *et al.*, 2012 ▸) to gossypol in cotton (Zhang *et al.*, 2019 ▸) and antioxidants. The key metabolites formed by the isoprenoid biosynthesis pathway are isopent­enyl pyrophosphate (IPP) and its isomer dimethylallyl isopentenyl pyrophosphate (DMAPP), which are fundamental building blocks of all isoprenoids.

**Figure 1:**
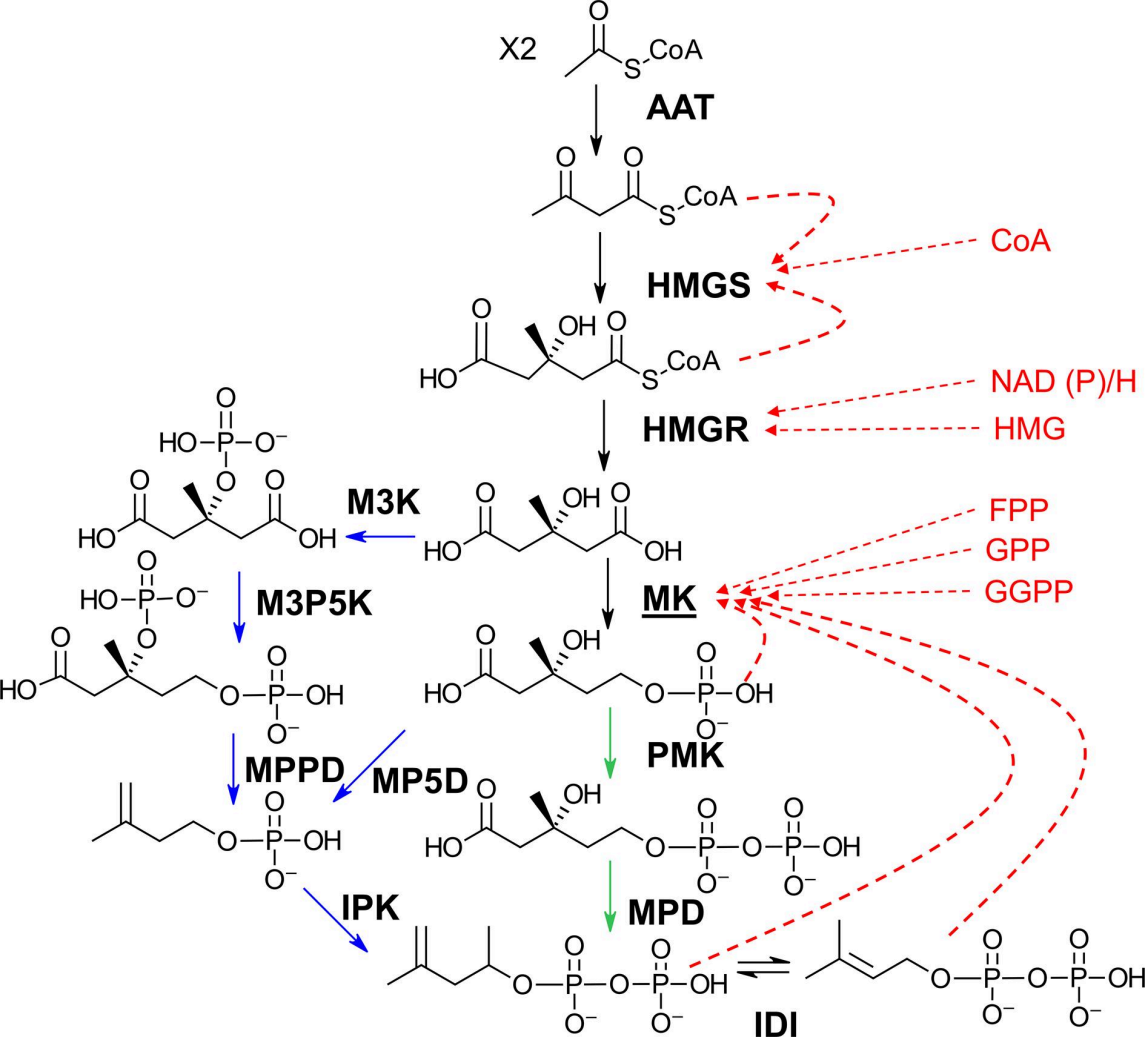
Upper mevalonate pathways in eukaryotes/eubacteria and archaea, showing the enzymes in bold. Blue arrows show enzymes that are found only in archaea, green arrows show reactions that are only present in eukaryotes/prokaryotes and red arrows show a non-exhaustive list of metabolites known to inhibit eukaryotic/prokaryotic enzymes. AAT, acetyl-CoA acetyltransferase; HMGS, hydroxymethylglutaryl-CoA synthase; HMGR, hydroxy­methylglutaryl-CoA reductase; MK, mevalonate kinase; M3K, mevalonate-3-kinase; M3P5K, mevalonate-3-phosphate-5-kinase; PMK, phosphomevalonate kinase; MPPD, mevalonate-3,5-pyrophosphate decarboxylase; MP5D, mevalonate-5-phosphate decarboxylase; MPD, mevalonate pyrophosphate decarboxylase; IPK, isopentenyl kinase; IDI, isopentenyl diphosphate isomerase; CoA, coenzyme A; HMG, hydroxymethylglutaryl; NADP, nicotinamide adenine dinucleotide phosphate; FPP, farnesyl pyrophosphate; GPP, geranyl pyrophosphate; GGPP, geranyl pyrophosphate synthase.

Two main pathways are known to lead to the production of IPP and DMAPP. One, the 2-*C*-methyl-d-erythritol 4-phosphate (MEP) pathway, is mainly found in eubacteria and photosynthetic organisms, while the other, the mevalonate (MVA) pathway, is mostly present in nonphotosynthetic eukaryotes and archaea (Fig. 1 ▸). Previous work on the archaeal MVA pathway revealed that this pathway lacks the three last enzymes of the eukaryotic MVA pathway: phosphomevalonate kinase (PMK), mevalonate pyrophosphate decarboxylase (MPD) and isopentenyl diphosphate isomerase (IDI). Grochowski *et al.* (2006 ▸) discovered a novel archaeal route to IPP while investigating the phosphomevalonate kinase pathway of the archaeon *Methanocaldococcus jannaschii* (Fig. 1 ▸). Since then, many more variations in the MVA pathway have been reported (Vinokur *et al.*, 2014 ▸, 2016 ▸; Yoshida *et al.*, 2020 ▸; Dellas *et al.*, 2013 ▸).

Another peculiarity of the MVA pathway is its regulatory mechanisms. The right panel of Fig. 1 ▸ presents the eukaryotic MVA pathway, which is known to be tightly regulated by metabolite-mediated feedback inhibition of the enzymes HMG synthase (HMGS), HMG reductase (HMGR) and mevalonate kinase (MK) (Chatzivasileiou *et al.*, 2019 ▸; Fu *et al.*, 2008 ▸), which together make up the upper mevalonate pathway. The left panel of Fig. 1 ▸ represents the archaeal pathway, in which most of the mevalonate kinases purified from archaea have been found to lack feedback inhibition (Kazieva *et al.*, 2017 ▸; Primak *et al.*, 2011 ▸), suggesting that the mevalonate pathway is regulated by a different mechanism in this kingdom.

Mevalonate kinases (MK) catalyse the phosphorylation of mevalonate. Many MKs have been characterized and studied, as they have been implicated in human diseases such as mevalonic aciduria, hyperimmunoglobulinemia D and periodic fever syndrome (Favier & Schulert, 2016 ▸). MKs are also potentially interesting drug targets, as they play essential roles in the metabolism of pathogenic organisms such as *Leishmania* (Shafi *et al.*, 2021 ▸; Prasad *et al.*, 2022 ▸) and in some eubacteria, for example *Streptococcus pneumoniae* (Kudoh *et al.*, 2010 ▸) and *Staphylococcus aureus* (Ferrand *et al.*, 2011 ▸). Finally, as they are generally highly regulated by feedback inhibition, alternative MK enzymes such as archaeal MKs are also interesting targets for the metabolic engineering of terpenes. For example, Chen and coworkers increased the production of lycopene by expressing a variant of *Saccharo­myces cerevisiae* MK in *Escherichia coli* (Chen *et al.*, 2018 ▸). A review by Rinaldi and coworkers also describes the use of an archaeal MK originating from *Methanosarcina mazei* (MKmaz) as a strategy to obtain a higher isoprene titre in *E. coli* (Rinaldi *et al.*, 2022 ▸).

Tardigrades are known for their ability to survive extreme environments. The genome of *Ramazzottius varieornatus* was sequenced in 2016 (Hashimoto *et al.*, 2016 ▸). Most of their incredible abilities, such as desiccating and rehydrating, for example, have been linked to tardigrade-specific proteins (Arakawa, 2022 ▸). However, the investigation of some more common proteins in tardigrades might still reveal interesting new features; therefore, we decided to characterize the mevalonate kinase from *R. varieornatus*.

Psychrophilic enzymes, although inherently less stable than their mesophilic and thermophilic counterparts, are used in laundry detergents and for bioremediation purposes, and are attracting increasing interest from other industries (Liu *et al.*, 2023 ▸). Indeed, psychrophilic enzymes may be more cost-effective and environmentally friendly by reducing the requirement for heating and reducing the concomitant risk of contaminating microorganisms, which generally require higher temperatures (Kumari *et al.*, 2021 ▸).

As of mid-2023, there were 13 structures of mevalonate kinase in the PDB originating from nine different species. As mevalonate kinases are so widely distributed, it is possible to identify homologous enzymes that catalyse the same reaction but have very different biochemical characteristics, inhibition profiles and temperature optima. This diversity represents an opportunity to further our understanding of the feedback inhibition of the enzyme or the lack thereof, as well as exploring the variations seen in the thermal tolerance of the enzyme. In recent years, only a few feedback-resistant archaeal MKs have been purified and characterized (Primak *et al.*, 2011 ▸; Kazieva *et al.*, 2017 ▸), including a thermophilic mevalonate kinase from *Methanocaldococcus jannaschii* (Yang *et al.*, 2002 ▸; Huang *et al.*, 1999 ▸). To date, there are no reports of the characterization of a psychrophilic MK enzyme. In 2004, Nichols and coworkers identified enzymes of the MVA pathway in the genome of the cold-adapted *Methanococcoides burtonii* by homology (Nichols *et al.*, 2004 ▸), but these enzymes have yet to be isolated and characterized. Having psychrophilic versions of known mesophilic and thermophilic homologues ultimately helps with understanding the cold-adaptation mechanisms and can lead to possible engineering strategies (Liu *et al.*, 2023 ▸; Kumari *et al.*, 2021 ▸).

In this work, we have identified, purified and characterized, both biochemically and structurally, two previously uncharacterized mevalonate kinases originating from the extremotolerant tardigrade *R. varieornatus* (Hashimoto *et al.*, 2016 ▸; MKvar) and the psychrophilic archaeon *M. burtonii* (MKbur; Allen *et al.*, 2009 ▸). As part of this study, the crystal structure of MKvar with mevalonate in the active site was determined to 2 Å resolution and the crystal structure of MKbur was determined to 2.2 Å resolution. Like other previously characterized archaeal MK enzymes (Kazieva *et al.*, 2017 ▸; Primak *et al.*, 2011 ▸), MKbur was found to be a typical feedback-resistant archaeal MK, whereas MKvar was found to be inhibited in the presence of prenylphopsphate geranyl pyrophosphate (GPP) and farnesyl pyrophosphate (FPP), as well as in the presence of its substrate/product in the 2 m*M* range.

## 2. Materials and methods

### 2.1. Cloning

After codon optimization for expression in *E. coli*, genes encoding the mevalonate kinase from *Methanosarcina mazei* (MKMaz) and the two putative mevalonate kinases from *Ramazzottius varieornatus* (MKvar; BDGG01000012.1, GAV05667.1) and *Methanococcoides burtonii* (MKbur; WP_011500381.1, Q12TI0) were ordered from Twist Bioscience (San Francisco, California, USA; Supplementary Fig. S1). The genes were cloned into a pBAD vector, adding a sequence encoding a 6×His tag to the N-terminus (Esquirol *et al.*, 2022 ▸). Subcloning was performed in DH5α cells (New England Biolabs, USA).

### 2.2. Protein expression

Vectors built using an acceptor vector (cassettes 5 and 6) producing His-tagged protein were used to transform *E. coli* BL21 (λDE3) cells (New England Biolabs, USA). Bacteria were grown on lysogeny broth (LB) medium containing 100 µg ml^−1^ ampicillin for the pBAD vectors at 37°C with shaking at 180 rev min^−1^ until OD_600_ reached 0.5. Induction was triggered by the addition of 0.2% l-arabinose and the temperature was decreased to 28°C. The cells were incubated overnight before harvesting by centrifugation at 5000*g* for 15 min using an Avanti J-E centrifuge (Beckman Coulter, Indianapolis, USA).

The temperature was kept at 4°C during all of the following purification steps. The cells were resuspended in buffer *A* (50 m*M* Tris, 100 m*M* NaCl, 5 m*M* imidazole pH 8) and lysed by passage through an Emulsiflex-C5 homogenizer (Avestin, Canada) five times at 137 MPa. Lysis was followed by centrifugation at 18 000*g* for 30 min to pellet the cellular debris. The soluble fraction was then filtered through a 0.22 µm syringe filter (Millipore, USA). The soluble fraction was loaded onto a HisTrap FF column (Cytivia) and eluted with buffer *B* (50 m*M* Tris, 100 m*M* NaCl, 500 m*M* imidazole pH 8) using an ÄKTApure (GE Healthcare Life Sciences). The purity of the sample was assessed by SDS–PAGE analysis on Mini-PROTEAN Precast Gels (Bio-Rad). Fractions containing the protein of interest were concentrated using an Amicon Ultra centrifugal filter and rinsed with a buffer consisting of 50 m*M* Tris, 100 m*M* NaCl pH 8. A final purification step was performed on a Superdex 200 10/300 GL size-exclusion column (GE Healthcare) equilibrated with 50 m*M* Tris, 100 m*M* NaCl pH 8. Final concentration of the proteins was performed using an Amicon Ultra centrifugal filter. The concentrated proteins (MKvar at 8.8 mg ml^−1^, MKbur at 6 mg ml^−1^ and MKmaz at 4.7 mg ml^−1^) were aliquoted in 100 µl tubes, snap-frozen in liquid nitrogen and kept in a freezer at −80°C until further use.

### 2.3. Mass spectrometry

To validate the sequence of His-tagged mevalonate kinase from *R. varieornatus* (UniProt Accession No. A0A1D1VW28), a gel band of purified MKvar was subjected to manual in-gel reduction, alkylation and tryptic digestion. The sample was reduced with 10 m*M* DTT (Sigma) for 30 min, alkylated for 30 min with 50 m*M* iodoacetamide (Sigma) and digested with 375 ng Trypsin Gold (Promega) for 16 h at 37°C. Extracted peptides were then analysed by matrix-assisted laser de­sorption ionization (MALDI) using an UltrafleXtreme MALDI-TOF mass spectrometer (Bruker). Equal volumes of tryptic peptide and matrix solutions were mixed and deposited onto ground-steel BC target plates (MTP 384, Bruker). External calibration was performed using Peptide Calibration Standard II (Bruker, *m*/*z* mass range 700–3500 Da) and MALDI-TOF spectra were searched against a decoy database containing sequences of the MKvar construct plus common proteomics contaminants using the *MASCOT* peptide mass fingerprint search engine (version 2.3; Matrix Science).

To confirm the accurate mass of MKvar, liquid chromatography–mass spectrometry (LC-MS) was carried out using an UltiMate 3000 HPLC system (Thermo Fisher Scientific) coupled to a maXis II Q-TOF mass spectrometer (Bruker), as described previously (Newman *et al.*, 2019 ▸). Briefly, a 10 ml aliquot of purified protein (1 mg ml^−1^) was loaded onto a 50 × 4.6 mm, 5 µm particle-size, 300 Å pore-size PLRP-S column (Agilent) pre-equilibrated with 0.1% formic acid. The protein was eluted from the column at a flow rate of 250 µl min^−1^ by applying a linear 30 min gradient from 0 to 80% mobile phase *B* [mobile phase *A*, 0.1%(*v*/*v*) formic acid; mobile phase *B*, 90%(*v*/*v*) acetonitrile/0.1%(*v*/*v*) formic acid] and ionized using an Apollo II electrospray ion source (Bruker) with the nebulizer pressure set to 1.8 bar and dry gas maintained at 220°C at a flow rate of 8 l min^−1^. High-resolution LC-MS data were analysed using the Protein Metrics *Intact Mass* parsimonious charge-state deconvolution algorithm (Bern *et al.*, 2018 ▸).

### 2.4. Structure determination of MKvar

MKvar protein (3 mg ml^−1^ in 50 m*M* Tris–HCl, 50 m*M* NaCl pH 8 with either 2 m*M* mevalonate or 2 m*M* ATP) was set up in initial crystallization screens at both 20 and 8°C. An initial hit which grew overnight [0.2 *M* calcium chloride, 28%(*v*/*v*) polyethylene glycol 400, 0.1 *M* sodium HEPES 7.5, protein and mevalonate] was used to seed into other PEG 400 conditions. Finally, additive screening experiments were set up around hits from the optimization step. Crystals grown from the additive screening showed diffraction limits from 6 Å to better than 2.5 Å. The best crystals contained protein treated with mevalonate and were harvested from drops containing 0.126 *M* calcium acetate, 24.9%(*v*/*v*) polyethylene glycol 400, 0.09 *M* sodium HEPES pH 8.4 with the additives Gly-Gly-Gly (0.03 *M*) or taurine (0.01 *M*) at 20°C. All crystallization trials were set up in SD-2 plates (SwissSci) with drops consisting of 150 nl protein solution and 150 nl reservoir solution equilibrated against 50 µl reservoir solution. Crystals were harvested by adding 1.3 µl reservoir solution to the crystal drop and then gently pulling the crystals out with a MiTeGen mylar loop. No additional cryoprotection was added as the growth conditions were sufficiently cryoprotecting.

Two data sets from the MX1 beamline at the Australian Synchrotron were processed using *XDS* (Kabsch, 2010 ▸) and *AIMLESS* (Evans & Murshudov, 2013 ▸) to give a total of 720° of data to higher resolution and merged as the unit-cell parameters were essentially the same; this gave a 2 Å resolution data set in space group *P*2_1_2_1_2_1_. All data sets were collected at 100 K, collecting 360° of data in 36 s.

The initial molecular-replacement (MR) solution obtained using PDB entry 2r42, *CHAINSAW* (Stein, 2008 ▸) and *Phenix* (Liebschner *et al.*, 2019 ▸) was poor. It was clear that there were two copies of the protein in the asymmetric unit and there was decent density for a portion of the structure near the N-terminus and then again near the C-terminus. Several mevalonate kinase structures were overlaid and common features were manually cut out to build significant portions of the structure using PDB entries 2r42, 1kkh, 1kvk, 2hfu, 4hac and 4rkz. After two rounds of this process, a model that was about 60% of the full-length protein gave a significantly better score in *Phenix*: a refined LLG of 248 and a TFZ of 17.8 (the previous values were an LLG of 111.9 and a TFZ of 12.8).

*Buccaneer* (Cowtan, 2006 ▸) was used to attempt to build a model into the density, but this failed. A model was built into the density manually with *Coot* (Emsley *et al.*, 2010 ▸) for four rounds of manual and *REFMAC* (Murshudov *et al.*, 2011 ▸; Agirre *et al.*, 2023 ▸) refinement before setting up *Phenix Autobuild* (Terwilliger *et al.*, 2008 ▸). *Phenix Autobuild* gave a more complete structure, which required another nine rounds of manual rebuilding (*Coot*) and refinement (*REFMAC*) to give a final structure.

*PDBeFold* (Krissinel & Henrick, 2004 ▸) showed that we did not choose the most structurally homologous available PDB entries for the initial MR. PDB entry 4ut4 has 17% sequence identity and an r.m.s.d. of 2.0 Å to our structure over 308/309 aligned amino acids, with a *Q*-score of 0.49 and a *Z*-score of ∼13.5. PDB entry 2r42 was 19th on the list, with a *Q*-score of 0.39, a *Z*-score of 9.9, 333 aligned residues and a sequence identity of 33% with an r.m.s.d. of 2.9 Å.

Using the *SSM* algorithm (Krissinel & Henrick, 2004 ▸) in *Coot* the numbers are slightly different: an r.m.s.d. of 3.05 Å over 335 aligned residues with 21 gaps and 34% sequence identity for PDB entry 2r42. When visualized, it is clear that the two structures have the same fold, but much of their secondary structures (particularly the helices and loops) are out of registration with each other, which was why it was a difficult MR solution.

For MKbur (6 mg ml^−1^), crystals were obtained from an optimization plate set up with 20% PEG 8000, 200 m*M* MgCl_2_, 100 m*M* Tris pH 8.5 with 3% trehalose as an additive. Well diffracting crystals that diffracted to about 2.2 Å resolution were obtained from several drops in this additive plate, so the additives were not the important factor here (they were all quite different). Crystals were obtained at both 4 and 20°C, so temperature was not particularly important in this case either. In both cases, MKvar and MKbur, the protein was flash-frozen in liquid nitrogen in small (100 µl) aliquots and defrosted just prior to crystallization trials.

An initial MR (*Phaser*; McCoy *et al.*, 2007 ▸) solution for MKbur was found in space group *P*2_1_ using the MKvar structure as a starting point. A solution for a single protomer was found; this was modified manually in *Coot* (Emsley *et al.*, 2010 ▸) and a second round of *Phaser* was then run to obtain two molecules in the asymmetric unit. The coordinates were rebuilt manually in *Coot* and were refined with *REFMAC* (Murshudov *et al.*, 2011 ▸; Agirre *et al.*, 2023 ▸).

The structures were validated using the tools in *Coot* and the final structures were verified using the validation tools from the RCSB PDB during the deposition process. Ramachandran statistics from the RCSB PDB are provided in Table 1 ▸. Coordinates and structure factors have been deposited in the PDB as entries 8tfo and 8teb.

### 2.5. Protein alignment

For sequence alignment, the *T-Coffee* server was used (Notredame *et al.*, 2000 ▸). All images were produced with *PyMOL* (version 1.8, Schrödinger; DeLano & Lam, 2005 ▸; DeLano, 2009 ▸) and the PDB entries for mevalonate kinases from *Homo sapiens* (PDB entry 2r3v; Fu *et al.*, 2008 ▸), *Rattus norvegicus* (PDB entry 1kvk; Fu *et al.*, 2008 ▸), *Staphylococcus aureus* (PDB entry 2x7i; Voynova *et al.*, 2004 ▸; Oke *et al.*, 2010 ▸), *Methanocaldococcus jannaschii* (PDB entry 1kkh; Huang *et al.*, 1999 ▸; Yang *et al.*, 2002 ▸), *Streptococcus pneumoniae* (PDB entry 2oi2; Andreassi *et al.*, 2007 ▸, 2009 ▸) and *Methanosarcina mazei* (PDB entry 6mde; Miller & Kung, 2018 ▸).

### 2.6. Enzyme assay

#### 2.6.1. Kinetics

(±)-Mevalonolactone was purchased from Sigma–Aldrich and dissolved in 50 m*M* Tris, 100 m*M* NaCl pH 8. Mevalonate was obtained by incubating mevalonolactone with potassium hydroxide in a 1:1 molar ratio for 2 h at 37°C before readjusting the pH to 8.

Reaction progress was monitored by following the NADH consumption at 340 nm with a FLUOstar Omega UV spectrophotometer (BMG Labtech) at 25°C, using a coupled assay relying on lactate dehydrogenase and pyruvate kinase. Overall, for each molecule of ADP produced by the reaction, one molecule of NADH will be oxidized by lactate dehydrogenase.

The coupled assay, adapted from previous publications (Kazieva *et al.*, 2017 ▸; Primak *et al.*, 2011 ▸; Huang *et al.*, 1999 ▸; Chu *et al.*, 2007 ▸; Fu *et al.*, 2008 ▸; Sgraja *et al.*, 2007 ▸), was performed using 0–5 m*M* mevalonate and a fixed nonlimiting ATP concentration of 5 m*M* or 0–5 m*M* ATP with a fixed nonlimiting concentration of 5 m*M* mevalonate, 0.4 m*M* phosphoenolpyruvate, 12 units of pyruvate kinase, 15 units of lactate dehydrogenase, 10 m*M* MgCl and 0.2 m*M* NADH.

The enzyme concentrations used in the assay ranged from 20 n*M* for MKmaz to 50 n*M* for MKbur and MKvar. Reactions were triggered by the addition of the substrate, either mevalonate or ATP. Kinetic data measurements of enzymes (*n* = 4) were used to calculate *K*_m_ and *k*_cat_ using *GraphPad Prism* 8 for Windows (GraphPad Software, La Jolla, California, USA)

#### 2.6.2. Inhibition and temperature dependence

The specific activity of each enzyme was measured in the presence of 100 µ*M* GPP or FPP and of 1.5 m*M* mevalonate and 3 m*M* ATP.

To test the temperature dependence, enzymes were assayed using the coupled assay described above in the presence of 1.5 m*M* mevalonate and 3 m*M* ATP after incubation for 5 min at a given temperature.

### 2.7. Differential scanning fluorimetry (DSF)

The three mevalonate kinases were each diluted to a final concentration of ∼0.07 mg ml^−1^ (1.5 µ*M*) in a final volume of 512 µl crystallization buffer (50 m*M* Tris–HCl, 50 m*M* NaCl pH 8.3); 8 µl 1:20 diluted SYPRO dye (Sigma) was added and 24 replicate wells for each protein were set up in a 96-well plate (Thermo AB-800 white PCR plate). Lysozyme at 0.02 mg ml^−1^ in the same buffer, with the same amount of SYPRO dye, was used as a control (also 24 replicate wells). The temperature was increased from 20 to 100°C in increments of 0.5°C every 5 s using a Bio-Rad CFX96 RtPCR machine. The results were viewed using the Bio-Rad *CFXmanager* software and the melting temperature was derived using *Meltdown* (Rosa *et al.*, 2015 ▸).

## 3. Results and discussion

### 3.1. Difference in thermostability of the mevalonate kinases

Two uncharacterized mevalonate kinase candidates were identified by using protein *BLAST* to search the genomes of the eukaryote *R. varieornatus* (Hashimoto *et al.*, 2016 ▸) and the psychrophilic archaeon *M. burtonii* (Allen *et al.*, 2009 ▸) using the sequences of MKmaz and rat (*Rattus norvegicus*) mevalonate kinase (MKrat; Fu *et al.*, 2002 ▸; Supplementary Fig. S1). The survival of extremophile organisms often relies on the production of isoprenoid derivatives; for example, in the case of *M. burtonii* an unusual lipid content in the membrane is thought to allow resistance to cold temperatures (Nichols *et al.*, 2004 ▸). Therefore, we sought to investigate the mevalonate kinases from these cold-adapted or cold-tolerant organisms.

The MK genes encoding MKvar and MKbur were ordered, cloned and expressed with a C-terminal 6×His tag for MKbur and MKmaz and an N-terminal 6×His tag for MKvar for purification by Ni–NTA and size-exclusion chromatography. Recombinant MK proteins were found to elute at around 180 m*M* imidazole for MKvar and at 100 m*M* imidazole for both MKbur and MKmaz (Supplementary Fig. S2).

**Figure 2:**
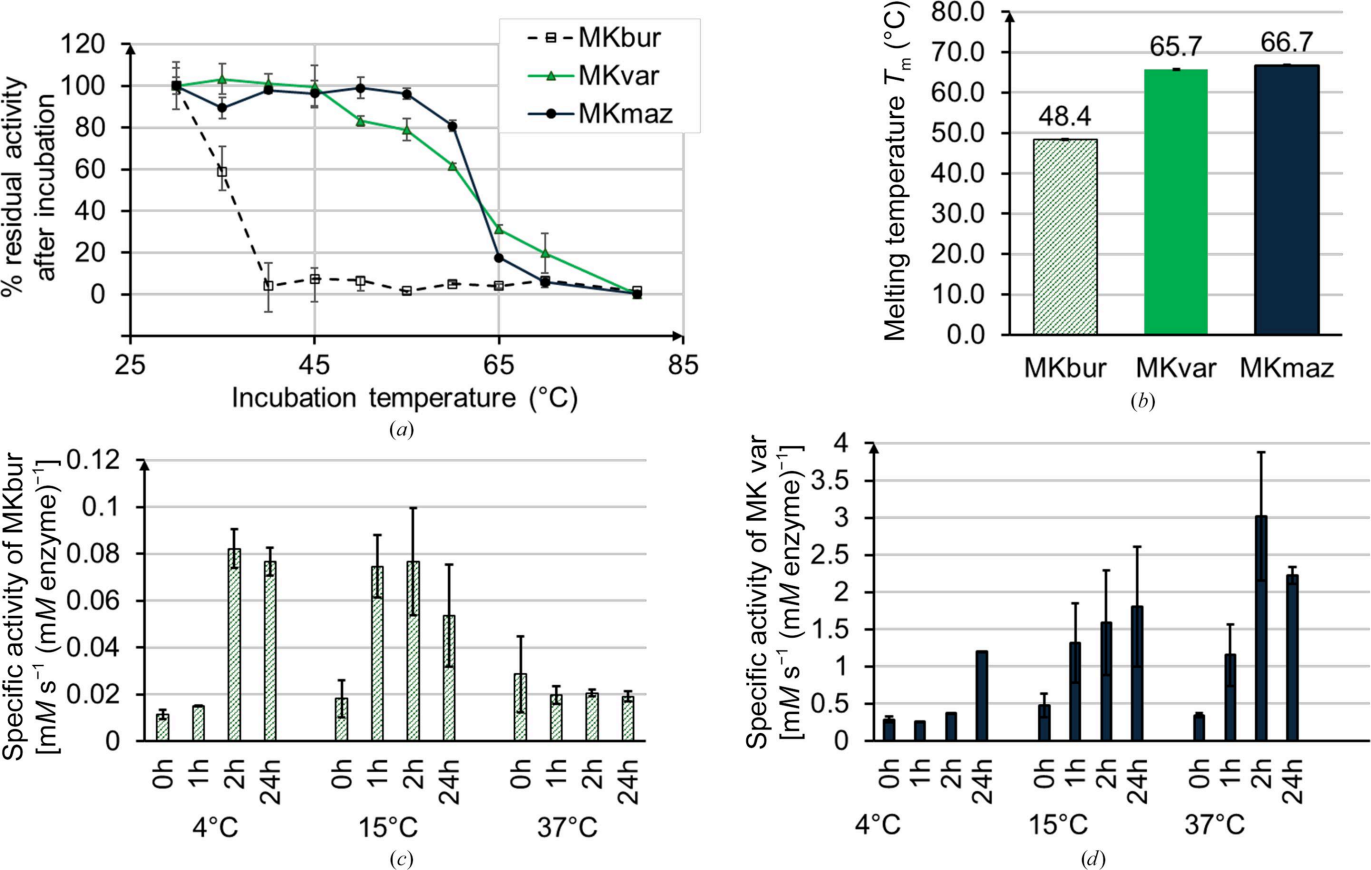
(*a*) Residual activity of the MKs after 5 min incubation at a given temperature as a percentage of the highest specific activity. (*b*) Differential scanning fluorometry showing the melting temperature of the protein. (*c*, *d*) Specific activity of (*c*) MKbur and (*d*) MKmaz after incubation for 0, 1, 2 and 24 h at 4, 15 and 37°C (*n* = 3).

Differential scanning fluorometry (DSF) shows melting temperatures of 48.4, 65.7 and 66.7°C for MKbur, MKvar and MKmaz, respectively (Fig. 2 ▸ *b*, Supplementary Fig. S3). The structures of the archaeal MKs MKmaz and MKbur are highly similar and their sequence alignment shows 61.1% identity and an r.m.s.d. of 0.77 Å (Supplementary Table S1); however, their thermostability assay and DSF results show a striking 20°C difference. Measuring residual activity after incubation at temperatures ranging from 25 to 80°C for 5 min revealed that the *T*_50_ values were 40°C for MKbur and 62°C for both MKvar and MKmaz (Fig. 2 ▸ *a*). The specific activity of MKmaz and MKbur incubated at 4, 15 and at 37°C was measured over time and while the highest specific activity for MKmaz was obtained at 37°C, MKbur appears to be most active at 15°C. Interestingly, in the case of MKbur activity can be detected with the same order of magnitude as in the 15°C samples after 2 h at 4°C (Fig. 2 ▸).

Chu *et al.* (2007 ▸) linked the thermostability of another archaeal MK, that from *Methanocaldococcus jannaschii*, to a disulfide bond linking a cysteine located in motif II and a cysteine located in motif III. A mutation of an alanine residue in motif III into a cysteine in MKrat was also shown to increase the thermostability of the enzyme. Although MKmaz, MKvar and MKbur possess the cysteine in motif III, MKmaz and MKvar lack the cysteine in motif II. It would be interesting to perform mutagenesis to assess its potential impact on thermostability.

In their review, Liu and coworkers list several characteristics of cold-activated enzymes: differences in the primary structure, such as proline-poor sequences, the presence of glycine, serine and histidine in the central domain, and differences in hydrogen bonding, where less bonding grants more flexibility; flexibility of the active site at the expense of binding efficiency is also reported (Feller & Gerday, 2003 ▸). Finally, enzymes from psychrophiles have fewer disulfide bonds, greater hydrophobicity and hydrophobic surfaces, and also appear to have a larger active-site cavity compared with mesothermophilic and thermophilic homologues (Liu *et al.*, 2023 ▸). Futher studies will be needed to understand the structural bases that allow MKbur to function at very low temperature.

### 3.2. Novel MKs display the conserved motifs of the GHMP kinase superfamily

**Figure 3:**
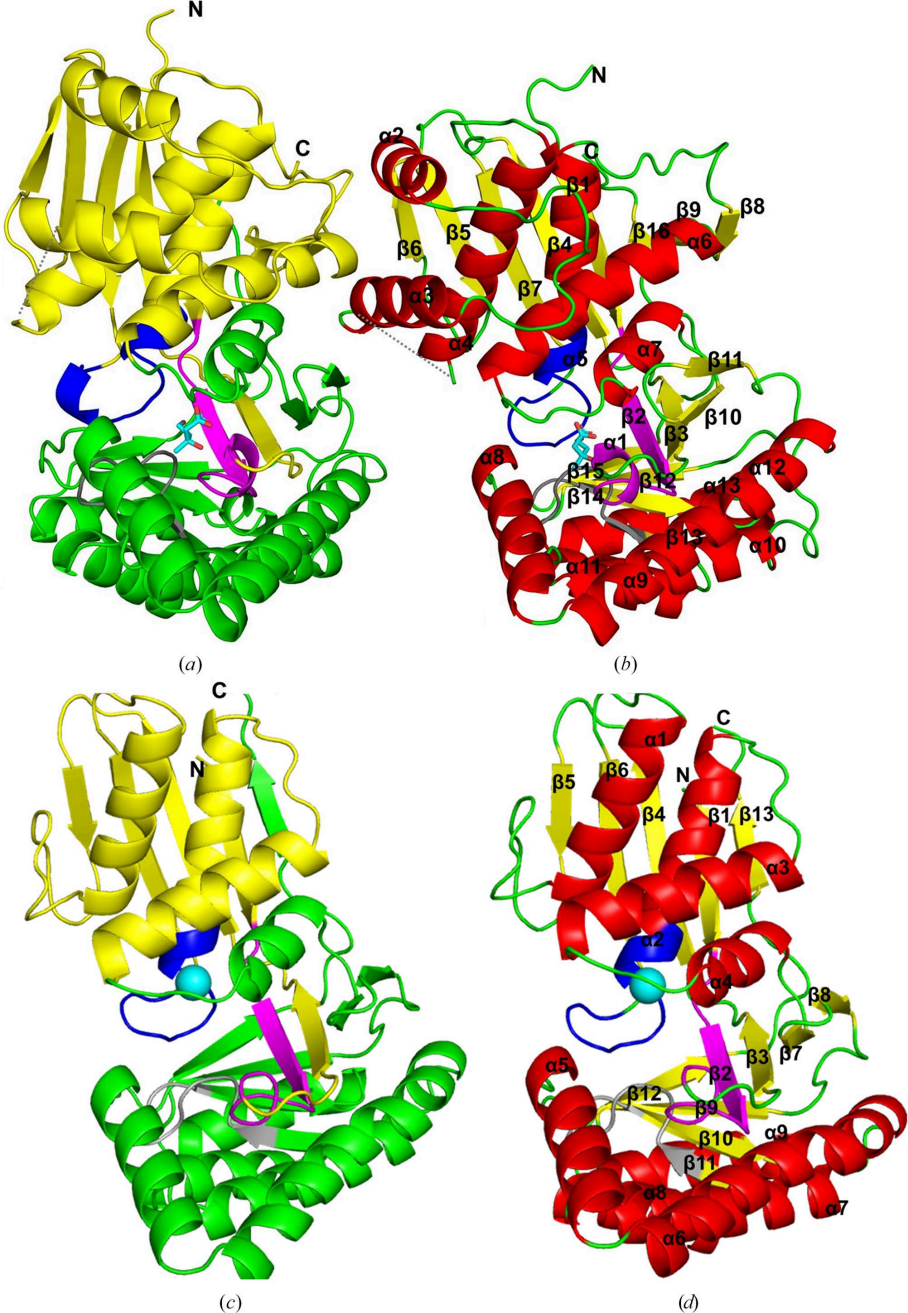
Cartoon representation of the monomers of (*a*, *b*) MKvar and (*c*, *d*) MKbur showing the N-terminal domains in yellow and the C-terminal domains in green (*a*, *c*) and with labelled helices in red, loops in green and β-sheets in yellow (*b*, *d*). An MVA molecule is shown as blue sticks (*a*, *b*) in the active site of MKvar; the locations of GHMP motifs I, II and III are indicated in pink, blue and grey, respectively, and the location of an Mg^2+^ ion involved in ATP binding is indicated by a cyan sphere in MKbur (*c*, *d*).

Mevalonate kinases belong to the GHMP kinase superfamily, which includes enzymes such as galactokinase, homoserine kinase, MK and phosphomevalonate kinase. The sequence alignment of MKmaz, MKrat, MKvar and MKbur is shown in Supplementary Fig. S4. Archaeal and eubacterial MK sequences tend to be shorter than those of eukaryotic MKs and differ in the N-terminal region of the enzyme, lacking two α-helices and the loop between them (Fig. 3 ▸, Supplementary Fig. S4). The C-terminus of the MKrat sequence is thought to be involved in metabolite-mediated allosteric inhibition of the enzyme, and it is notable that the eukaryotic sequence does not align with the C-termini of the feedback-resistant archaeal proteins (MKmaz and MKbur; Fu *et al.*, 2008 ▸; Supplementary Fig. S5).

We confirmed the identity of MKvar before crystallization and structural characterization using MALDI-TOF analysis of a tryptic digest. The sample was confidently identified as a tardigrade mevalonate kinase (UniProt Accession No. A0A1D1VW28) with 62% sequence coverage (Supplementary Fig. S7). Intact mass analysis by LC-MS suggested that the protein was very pure, with a major mass of 45 277.0 Da, corresponding to the theoretical mass of His-tagged MKvar. A mass addition of 178 Da suggested gluconoylation of the His tag. *E. coli* has been previously reported to (phospho)gluconoylate sequences (Oke *et al.*, 2010 ▸; Supplementary Fig. S7).

As expected, the sequences contain the conserved motifs of the GHMP enzyme superfamily (Yang *et al.*, 2002 ▸; Andreassi & Leyh, 2004 ▸; Fu *et al.*, 2002 ▸). Motif I is shown in pink in Fig. 3 ▸ and Supplementary Fig. S5. The second conserved GHMP motif is motif II, which is shown in blue (Fig. 3 ▸, Supplementary Fig. S5); this motif forms a loop that is involved in ATP binding (–PXGXGLGSSAA–). Finally, motif III, which is shown in grey (Fig. 3 ▸, Supplementary Fig. S5), is a conserved region which forms a glycine-rich loop and is known to be involved in substrate binding. The aspartate, which is the main catalytic residue (as determined by mutagenesis studies; Potter & Miziorko, 1997 ▸), is identified with a star, and other conserved amino acids involved in stabilizing the substrates are marked by arrows (Fu *et al.*, 2002 ▸; Andreassi & Leyh, 2004 ▸; Potter & Miziorko, 1997 ▸; Supplementary Fig. S5).

The data-collection and refinement statistics for the MKvar and MKbur crystal structures can be found in Table 1 ▸. The His tag was not resolved in the structure. Like all GHMP superfamily enzymes, each monomer is composed of two domains: an N-terminal domain and a C-terminal domain separated by a cleft (Fig. 3 ▸ *a*). Both the mevalonate-binding (motif I and III) and the ATP-binding (motif II) sites are located in the cleft between the two domains, where the mevalonate molecule can be seen (Fig. 3 ▸ *b*). Motif I is found in β2 in both MKs. Motif II is located in a loop between β7 and α5 in MKvar and between β6 and α2 in MKbur; it is identical in all of the enzymes from the superfamily and is not thought to rearrange upon binding to ATP (Andreassi *et al.*, 2007 ▸; Fig. 3 ▸). Finally, motif III is in a loop between β13 and β14 in MKvar and β10 and β11 in MKbur. Andreassi and coworkers describe the loop (between β12 and α8 in MKvar and between β9 and α5 inr MKbur) as a mobile section that forms a lid on the active site that protects MVA and ATP from solvent (Andreassi *et al.*, 2007 ▸; Fig. 3 ▸). The catalytic aspartate residue is found at the N-terminus of α7 in MKvar and the N-terminus of α4 in MKbur; this residue is highly conserved across the GHMP family, and although these enzymes act on different substrates and have different regulatory mechanisms (bi-bi, allostery), the involvement of the aspartate residue in the phosphorylation reaction is conserved (Roy *et al.*, 2019 ▸).

A highly conserved threonine residue at the N-terminus of the α8 helix of MKvar/the α5 helix of MKbur (Figs. 3 ▸ and 4 ▸ *b*) was also found to be involved in ATP and mevalonate binding, and its mutation led to an increase in *K*_m_ and a decrease in *k*_cat_ (Cho *et al.*, 2001 ▸). This mutation is also linked to the development of pathology in humans (Andreassi *et al.*, 2007 ▸).

A loop region between α3 and α4 in MKvar is not seen in the X-ray structure, suggesting high mobility of this loop; mobility in this region is also observed in some other MK structures (Fu *et al.*, 2008 ▸). The main structural differences observed between the MKs are found in the N-terminal domain, mirroring what is seen in the sequence alignment. The N-terminal sections missing from eubacterial and archaeal MKs mostly form the two helices α2 and α3 in MKvar and can also be found in rat and human MK (Fu *et al.*, 2008 ▸).

The MK structures were run through *PDBeFold* and the closest matches can be found in Supplementary Table S1, arranged by *Q*-score. Interestingly, the most structurally homologous match to MKvar was not a mevalonate kinase but was PDB entry 4ut4, the heptokinase WcbL from *Burkholderia pseudomallei* (Vivoli *et al.*, 2015 ▸), which was followed by a range of archaeal and eubacterial MKs and a phospho­mevalonate kinase from *Streptococcus pneumoniae*. MKbur returned very high similarity to MKs from archaeal origin, with the highest *Q*-scores of 0.9 for MK from *Methanosarcina mazei* and 0.6 for MK from *Methanocaldococcus jannaschii.*

### 3.3. Functional characterization of MKvar and MKbur

Steady-state kinetic values for (*RS*)-mevalonate and ATP were determined using a coupled enzyme assay as used previously (Primak *et al.*, 2011 ▸; Vinokur *et al.*, 2014 ▸; Fu *et al.*, 2008 ▸) and apparent kinetic parameters are given in Table 2 ▸; kinetic values for previously published mevalonate kinases are given in Table 3 ▸. Phosphatidic compounds are common inhibitors of mevalonate kinases (Fu *et al.*, 2008 ▸; Nyati *et al.*, 2015 ▸; Andreassi *et al.*, 2007 ▸). The MK from *Staphylococcus aureus* (MKStaph) was reported to be inhibited in the presence of its product mevalonate 5-phosphate at 1.56 m*M* (Voynova *et al.*, 2004 ▸; Table 3 ▸). Voynova and coworkers determined that the product inhibition of MKStaph was a sign of an ordered sequential mechanism involving first the binding of mevalonate before ATP and then the release of the product before the release of ADP (Voynova *et al.*, 2004 ▸). MKvar was found to be inhibited in the presence of >2 m*M* mevalonate substrate. Although IC_50_ and *K*_i_ are not directly comparable, globally the range of sensitivity is as follows: the most sensitive are the mammalian and mosquito MKs, inhibition of which by phosphatidic compounds is within the 10^−8^–10^−7^ *M* range, then yeast MKs, which are inhibited at around 10^−6^ *M*, followed by eubacterial MKs and *Methanocaldococcus jannaschii* MK, which are inhibited in the 10^−5^–10^−3^ *M* range (Table 3 ▸); finally, inhibition is undetected in the archaeal MKs.

**Figure 4:**
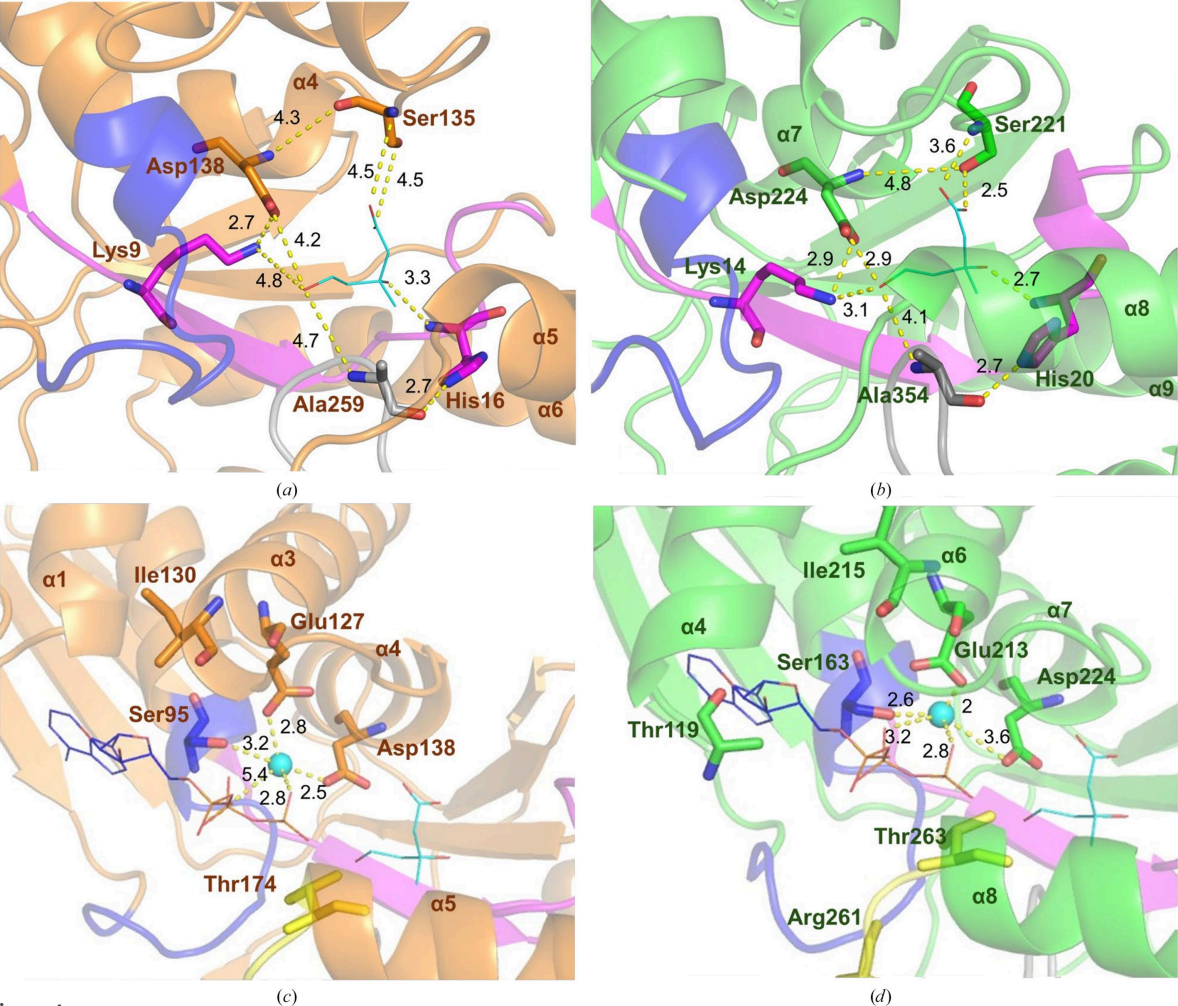
Zoom in around the active sites of MKbur in orange and MKvar in green, where (*a*) and (*b*) are centred around the mevalonate shown in cyan lines and (*c*) and (*d*) are centred around the ATP shown in lines and the magnesium ion (Mg^2+^) represented as a cyan sphere (the localization of ATP is estimated by alignment with MKrat crystallized with ATP in the active site; Fu *et al.*, 2002 ▸). Conserved amino acids are shown as sticks, with motifs I, II and III in pink, blue and grey, respectively. The loops involved in binding the nucleotide moiety are indicated in dark red and the lid is shown in yellow in (*c*) and (*d*). Distances in Å between elements are represented by yellow dotted lines.

The active sites of MKvar and MKbur are highly conserved compared with the active sites of the other characterized MKs (Fig. 4 ▸); all of the conserved amino acids are shown in sticks for MKvar and align with those in MKbur, as well as the motifs I, II and III characteristic of the GHMP family.

**Figure 5:**
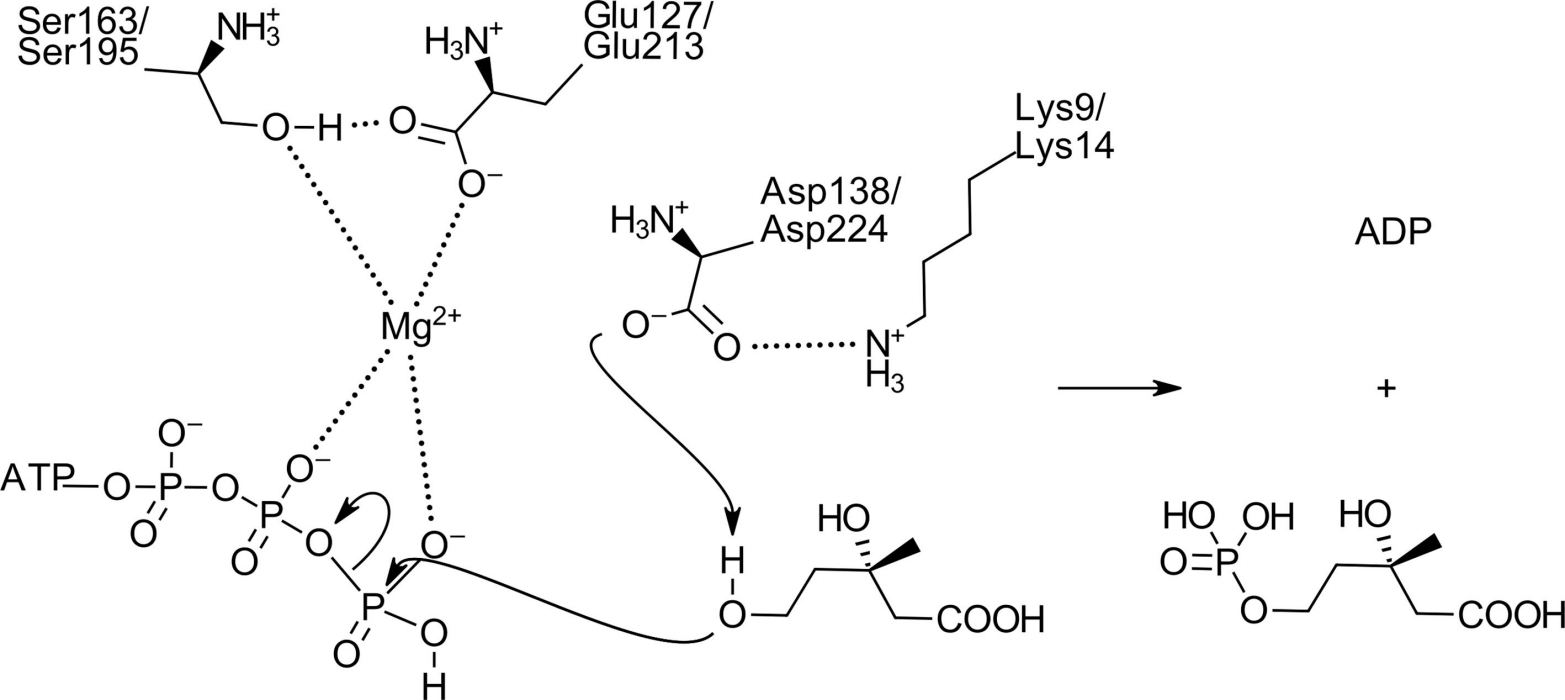
Scheme of the mevalonate kinase mechanism that converts mevalonate and ATP into mevalonate 5-phosphate and ADP, where the amino-acid numbering represents the sequences of MKbur/MKvar.

The C5 hydroxyl group of the mevalonate substrate is thought to be deprotonated by the catalytic aspartate residue, which then proceeds to perform a nucleophilic attack on the γ-phosphate of ATP (Fig. 5 ▸).

The mevalonate molecule is stabilized by interactions with the conserved amino acids located near the catalytic amino acid Asp224/Asp138 (in MKvar/MKbur). The catalytic role of this aspartate residue was demonstrated by Potter and Mizioro, who showed that mutation of the aspartate to an alanine or an arginine led to no detectable activity (Potter & Miziorko, 1997 ▸). The roles of Ser221/Ser135, His20/His16, Lys14/Lys9 and Ala354/Ala259 in MKvar/MKbur have been deduced from their conservation amongst the GHMP family as well as by their close location to the substrate (Sgraja *et al.*, 2007 ▸; Huang *et al.*, 2016 ▸; Miller & Kung, 2018 ▸; McClory *et al.*, 2019 ▸; Figs. 4 ▸ *a* and 4 ▸ *b*).

The highly conserved Glu213/Glu127 and Ser163/Ser95 (in MKvar/MKbur) have been found to be involved in ATP binding and more specifically to coordinate the Mg^2+^ ion that activates the γ-phosphate of ATP (Yang *et al.*, 2002 ▸; Fu *et al.*, 2002 ▸; Figs. 4 ▸ *c* and 4 ▸ *d*); when this Glu was mutated to an alanine by Potter and Mizioro it turned the human MK into a labile protein, and when it was replaced by a glutamine it lowered the *K*_m_ for ATP and the *V*_max_ (Potter & Miziorko, 1997 ▸). The conserved Thr residue involved in binding the tail of the ATP can be seen in the yellow lid portion (Thr263/Thr174 in MKvar/MKbur; Figs. 4 ▸ *c* and 4 ▸ *d*; Cho *et al.*, 2001 ▸). A conserved Arg residue in MKrat (Arg241), corresponding to Arg261 in MKvar, was found to be essential in binding the tail of ATP and moves inside the active site upon binding the substrates (McClory *et al.*, 2019 ▸); no equivalent arginine residue can be found in MKbur or MKmaz, where it is replaced by a serine.

### 3.4. Sensitivity to inhibition

**Figure 6:**
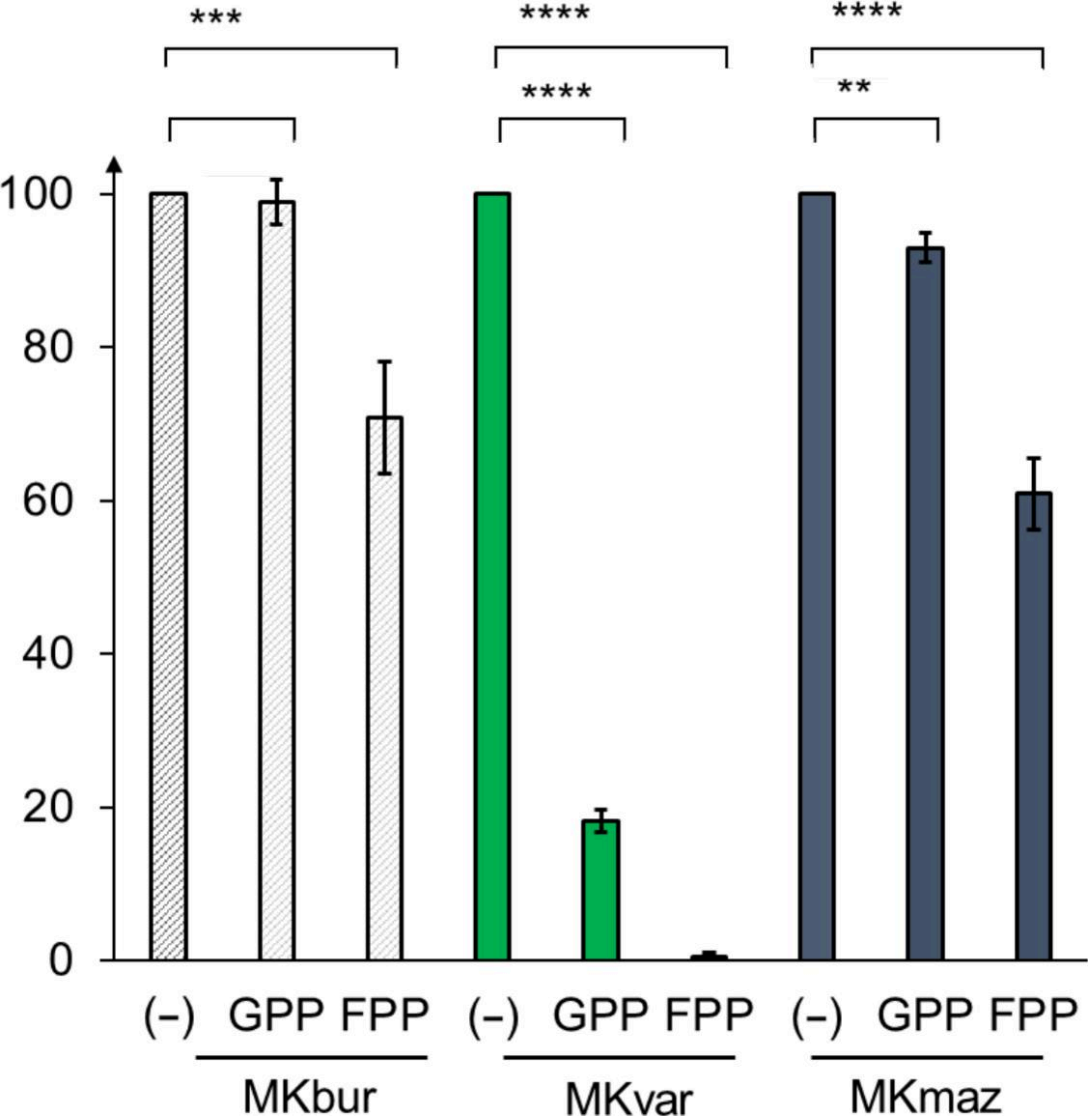
Residual activity of each mevalonate kinase measured in the presence of 100 µ*M* GPP or FPP (*n* = 3), where * indicates *P* ≤ 0.05, ** indicates *P* ≤ 0.01, *** indicates *p*-value ≤ 0.001 and *** indicates *p*-value ≤ 0.0001.

MKs are inhibited as part of the terpene pathway regulatory mechanism, usually by longer prenylphosphates built by the condensation of IPP/DMAPP in the lower part of the pathway (Potter & Miziorko, 1997 ▸); however, there is a range of sensitivity depending on the genus of origin (Table 3 ▸). In general, longer prenylphosphate compounds, such as FPP, appear to cause greater inhibition than the shorter GPP, which agrees with our observations (Fig. 6 ▸).

MKmaz and MKbur were found to possess activity in the presence of up to 100 µ*M* of both FPP and GPP, compared with the almost total loss of activity recorded for MKvar, where <20% residual activity remains in the presence of 100 µ*M* GPP and only 0.4% remains with 100 µ*M* FPP (Fig. 6 ▸). Kazieva *et al.* (2017 ▸) did not observe any inhibition of MKmaz activity in the presence of 100 µ*M* GPP and FPP, whereas we recorded a loss of activity, with 92% and 70% residual activity remaining in the presence of 100 µ*M* GPP and FPP, respectively (Fig. 6 ▸). This decrease might not be a reflection of inhibition; it might simply be a reflection of the thermodynamics of the reaction, or it may be explained by the difference in assay methods. MKbur, like MKmaz, appears to be less sensitive to inhibition.

**Figure 7:**
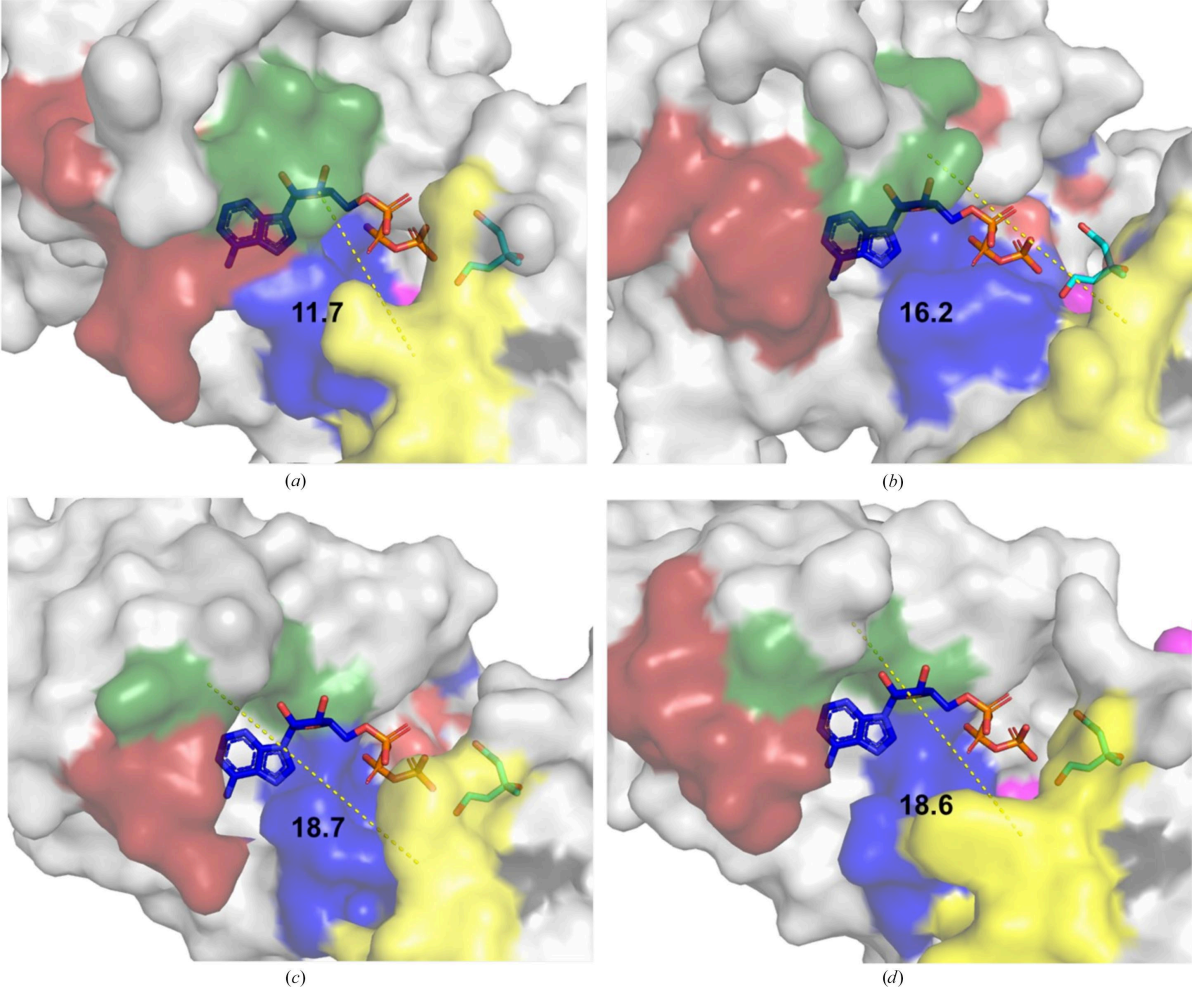
Surface representation and surface of (*a*) MKvar, (*b*) MKrat, (*c*) MKmaz and (*d*) MKbur with mevalonate and ATP shown as sticks. The yellow portion is the lid, blue represents motif II and dark red represents a variable nucleotide-binding loop; the dark green section represents the end of helix α4/α1 (in MKvar/MKbur) or the equivalent in MKrat and MKmaz. The measurement represents the distance in Å between the N-terminus of helix α4/α1 (in MKvar/MKbur) and the loop lid. The localization of ATP is estimated by alignment with MKrat crystallized with ATP in the active site.

The N-terminal end of helix α4/α1 (in MKvar/MKbur), marked in dark green in Fig. 7 ▸ and known to be involved in inhibition, also varies between feedback-inhibited MKvar and MKbur. The structural basis of the inhibition of MKs has been studied using the MKs from rat and human (Fu *et al.*, 2008 ▸). Fu and coworkers determined by mutagenesis that truncating the C-terminal end of human and rat MK (at Arg388, which does not exist in MKvar), and also mutating Thr104 and Ile196 in human MK, decreased the feedback inhibition in human MK (Fu *et al.*, 2008 ▸). In MKvar, Thr119 and Ile215 align with Thr104 and Ile196, respectively, in human MK. MKbur has a conserved Ile residue; however, Thr119, which can be found at the N-terminus of the α4 helix (Figs. 4 ▸ *c* and 4 ▸ *d*) in MKvar, is not present in the N-terminal region of α1 in MKbur. The distance between Thr119 (the N-terminus of the α4 helix) and the ribose of the ATP molecule is ∼3 Å in MKvar, compared with a distance of ∼7 Å to the N-terminus of the α1 helix in MKbur.

Comparison of the active sites of MKvar, MKrat, MKbur and MKmaz shows differences in the cleft region (Fig. 7 ▸, Supplementary Fig. S8 and Table S2). The sides of the cleft are composed of two variable regions: a variable loop (in dark red in Figs. 7 ▸, 4 ▸ *c* and 4 ▸ *d*) and the N-terminus of helix α4/α1 on one side (in MKvar/MKbur; dark green in Fig. 7 ▸) and a lid portion at the bottom on the other side (in yellow in Figs. 7 ▸, 3 ▸ *c* and 3 ▸ *d*). Further mutational studies would be needed in order to further specify the structural basis of inhibition of MKvar for prenylphosphate compounds or non-inhibition in the case of MKbur.

## 4. Conclusion

Extremophiles are actually often polyextremophiles, as an extreme environment can be cold but may often also involve high pressure or high salinity, for example. Therefore, they represent an untapped potential source of enzymes with unusual properties that are waiting to be investigated. This work describes the purification and characterization of two mevalonate kinases from *M. burtonii* and *R. varieornatus*. The structure of MKvar was solved at a resolution of 2 Å with mevalonate in the active site and the structure of MKbur was solved at a resolution of 2.2 Å. Both of these MKs appear to present highly conserved characteristics of the GHMP family. These two enzymes vary with regard to thermostability and their inhibition profile. MKbur, as expected from its psychrophilic origin, appears to conserve activity at low temperature and has relatively low thermostability. MKvar presents a classic feedback-inhibited profile, as well as inhibition by its own product, while MKbur, similar to many different archaeal MKs, does not appear to be significantly inhibited by FPP or GPP compounds.

## 5. Related literature

The following references are cited in the supporting information for this article: Hartley *et al.* (2004 ▸) and Mackinnon *et al.* (2021 ▸).

## Supplementary Material

PDB reference: mevalonate kinase from *Methanococcoides burtonii*, 8teb

PDB reference: from *Ramazzottius varieornatus*, 8tfo

Supplementary Figures and Tables. DOI: 10.1107/S2059798324001360/jb5061sup1.pdf

## Figures and Tables

**Table 1:** X-ray data-collection and refinement statistics for MKvar and MKbur Values in parentheses are for the highest resolution shell.

	MKvar	MKbur
PDB code	8tfo	8teb
Data collection		
Space group	*P*2_1_2_1_2_1_	*P*2_1_
*a*, *b*, *c* (Å)	45.1, 80.7, 207.5	38.3, 93.0, 90.4
α, β, γ (°)	90, 90, 90	90, 90.04, 90
Temperature (K)	100	100
Resolution (Å)	45.1–2.00 (2.05–2.00)	46.5–2.20 (2.26–2.20)
*R*_merge_ †	0.292 (4.656)	0.113 (0.717)
*R*_p.i.m._	0.057 (0.900)	0.046 (0.285)
CC_1/2_ †	0.999 (0.623)	0.998 (0.849)
〈*I*/σ(*I*)〉	11.8 (1.0)	13.7 (2.8)
Completeness (%)	99.8 (97.5)	99.5 (97.9)
Multiplicity	27.1 (26.8)	7.0 (7.0)
Refinement		
Resolution (Å)	44.1–2.00	46.5–2.20
Unique reflections	49698	30725
Completeness (%)	99.9	99.4
*R*_work_/*R*_free_ (%)	20.8/24.7	18.6/23.8
No. of atoms		
Total	6095	4619
Protein	5943	4414
Metal	1	2
Ligand	20	0
Waters	151	202
*B* factors (Å^2^)		
Overall	41.4	34.0
Protein (chain *A*/*B*)	45.1/42.2	31.7/40.8
Metal (Ca/Mg)	48.8	41.0
Ligand	34.4	
Water	37.9	32.9
R.m.s. deviations		
Bond lengths (Å)	0.006	0.007
Bond angles (°)	1.262	1.289
Ramachandran plot statistics		
Favoured (%/residues)	98/754	97/589
Outliers (%/residues)	0.1/1	0/0

† As defined by *AIMLESS* in the *CCP*4 suite of programs.

**Table 2:** Apparent steady-state kinetic constants of mevalonate kinases with mevalonate (MVA) as the substrate (*n* = 4)

Organism	*K*_m_, MVA/ATP (µ*M*)	*k*_cat_, MVA (s^−1^)	*k*_cat_/*K*_m_, MVA (s^−1^ m*M* ^−1^)
*Methanococcoides burtonii*	55 ± 11/331 ± 63	5 ± 0.1	90
*Methanosarcina mazei*	123 ± 9/511 ± 48	10 ± 0.1	81
*Ramazzottius varieornatus*	237 ± 68/4844 ± 1413	5 ± 0.8	21

**Table 3:** Kinetic constants and inhibition profiles for various mevalonate kinases MVA, mevalonate; PP-MVA, pyrophosphate mevalonate; GPP, geranyl pyrophosphate; FPP, farnesyl pyrophosphate; 5-P-MVA, mevalonate 5-phosphate.

Organism	Kingdom	*K*_m_, MVA/ATP (µ*M*)	*k*_cat_, MVA (s^−1^)	*k*_cat_/*K*_m_ (s^−1^ m*M* ^−1^)	*K*_i_ (µ*M*)	References
Human	Eukaryotes	40.8/178	ND	ND	0.035 (FPP)	Fu *et al.* (2008 ▸)
*Rattus norvegicus*	Eukaryotes	35/950	22	628	0.35 (FPP)	Chu & Li (2003 ▸), Fu *et al.* (2008 ▸), Voynova *et al.* (2004 ▸), Chu *et al.* (2007 ▸)
*Aedes aegypti*	Eukaryotes	90/140	10	111	0.55 (GPP), 0.44 (FPP)	Nyati *et al.* (2015 ▸)
*Saccharomyces cerevisiae*	Eukaryotes	131/650	38	290	2.3 (GPP), 1.9 (FPP)	Primak *et al.* (2011 ▸)
*Staphylococcus aureus*	Eubacteria	41/339	ND	ND	1560 (5-P-MVA), 46 (FPP)	Voynova *et al.* (2004 ▸), Oke *et al.* (2010 ▸)
*Streptococcus pneumoniae*	Eubacteria	236/372	11	47	>100 (FPP), PP-MVA†	Primak *et al.* (2011 ▸), Andreassi *et al.* (2007 ▸)
*Methanocaldococcus jannaschii*	Archaea	68.5/92	28.5	419	45% (10 µ*M* GPP)‡, 35% (10 µ*M* FPP)‡	Huang *et al.* (1999 ▸), Chu *et al.* (2007 ▸)
*Methanosaeta concilii*	Archaea	17/74	14	823	Not detected ‡	Kazieva *et al.* (2017 ▸)
*Methanocella paludicola*	Archaea	15/119	7	466	Not detected ‡	Kazieva *et al.* (2017 ▸)
*Nitrosopumilus maritimus*	Archaea	461/1006	12	26	Not detected ‡	Kazieva *et al.* (2017 ▸)

† Activity was detected.

‡ Percentage residual activity.
