# Supplementary material for: Characterization of novel mevalonate kinases from the tardigrade Ramazzottius varieornatus and the psychrophilic archaeon Methanococcoides burtonii
Source: Acta Crystallogr D Struct Biol. 2024 Feb 27;80(Pt 3):203–15. doi: 10.1107/S2059798324001360 (PMC10910542; doi:10.1107/S2059798324001360)
Supplement: Supplementary file 1 [file d-80-00203-sup1.pdf]

Acta Cryst D STRUCTURAL BIOLOGY

Volume 80 (2024)

Supporting information for article:

Characterization of novel mevalonate kinases from the tardigrade *Ramazzottius varieornatus* and the psychrophilic archaeon *Methanococcoides burtonii*

Lygie Esquirol, Janet Newman, Tom Nebl, Colin Scott, Claudia Vickers, Frank Sainsbury and Thomas S. Peat

1. Mevalonate kinase (MKmaz) from *Methanosarcina mazei* (WP_011033702.1, Q8PW39)

MVSCSAPGKIYLFGEHAVVYGETAIACAVELRTRVRAELNDSITIQSQIGRTGLDFEKHPYVS
AVIEKMRKSIPINGVFLTVDSDIPVGSGLGSSAAVTIASIGALNELFGFGLSLQEIAKLGHEIEI
KVQGAASPTDTYVSTFGGVVTIPERRKLKTPDCGIVIGDTGVFSSTKELVANVRQLRESYPD
LIEPLMTSIGKISRIGEQLVLSGDYASIGRLMNVNQGLLDALGVNILELSQLIYSARAAGAFG
AKITGAGGGGCMVALTAPEKCNQVAEAVAGAGGKVTITKPTEQGLKVD*LVPRGSLE*HHHH
HH

2. Mevalonate kinase (MKbur) from *Methanococcoides burtonii* (WP_011500381.1, Q12TI0)

MITCSAPGKVYLFGEHAVVYGEPAICCAVDIRTRVTVSPADTITISSSLGTTGIDFEVHPYVSA
VLERFQDISSFDGVDLRISSDIPVGSGLGSSAAVTVATIKAMDTLLDLGLELDDIAKMGHEVE
QNIQGTASPTDTYVCTMGGVVLIPQRKKLELIDCGILIGNTNIFSSTKELVGNVADLNERFPD
VVGPVLSSIGKLSVIGEGLVNDRDYVSVGELMNIDQGLLDAIGVSCAELSSLIYAARESGAY
GSKITGAGGGGCMVAISPRENVDSVAEAIGMAGGKVVVANATDIGVRVECQ*LVPRGSLE*HH
HHHH

3. Mevalonate kinase (MKvar) from *Ramazzottius varieornatus* (BDGG01000012.1, GAV05667.1)

MHHHHHHGSMSNLRHLRVSAPGKIILHGEHAVVYQKTAVALSLGLRTRLDLTETTDGRISII
MDKFLQHTSWSVEELSKIIDKVKIDANNPETELDQELVEDLRMMTSGHHYQNGDGPAVGN
PQAYSTQSVALVGFLYILVKLCKFSGKQRPPSIQISISSDIAISAGLGSSAAFAVCLSASLLSYL
GIIVCDRKNCADVDGKLVPSADQLALINHWAFMVEKIVHGSASGVDNAVSTYGGSIKYRNN
ELTRIGSGLKLDVLIVDTHVQRDTKKMLDIVRHRRKLYPAITNPVLEAIDGISETSSKILQHG
DGLPTGEEYEVIADLVRMNQNLLSTLGVSHPKLDVICETASRFGQAGKLTGAGGGGCAIVV
LDPDMRQFEHLRESIIAEYRRMEFKPHLAELGGPGVLFHPVPGS

Figure S1 List of the protein expressed in this work with accession number and Uniprot codes in brackets underlined are the his-tag and a glycine-serine linker, in italic thrombin cut.

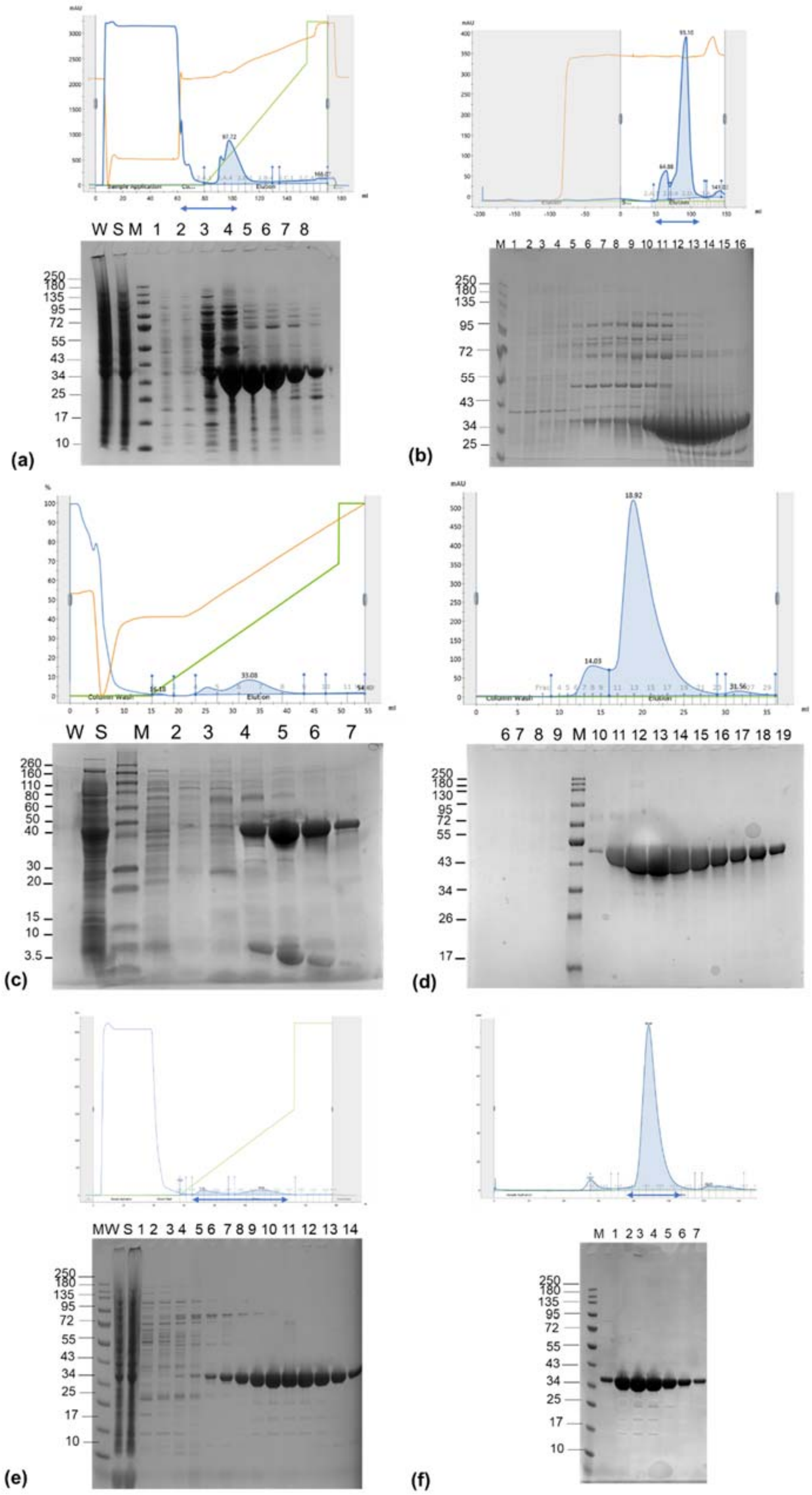

Figure S2 Traces (top) and corresponding SDS-PAGE analysis (bottom) for (a) NiNTA MKbur, (b) Size Exclusion MKbur, (c) NiNTA for MKvar, (d) Size Exclusion MKvar, (e) NiNTA for MKmaz, (f) Size Exclusion MKmaz.

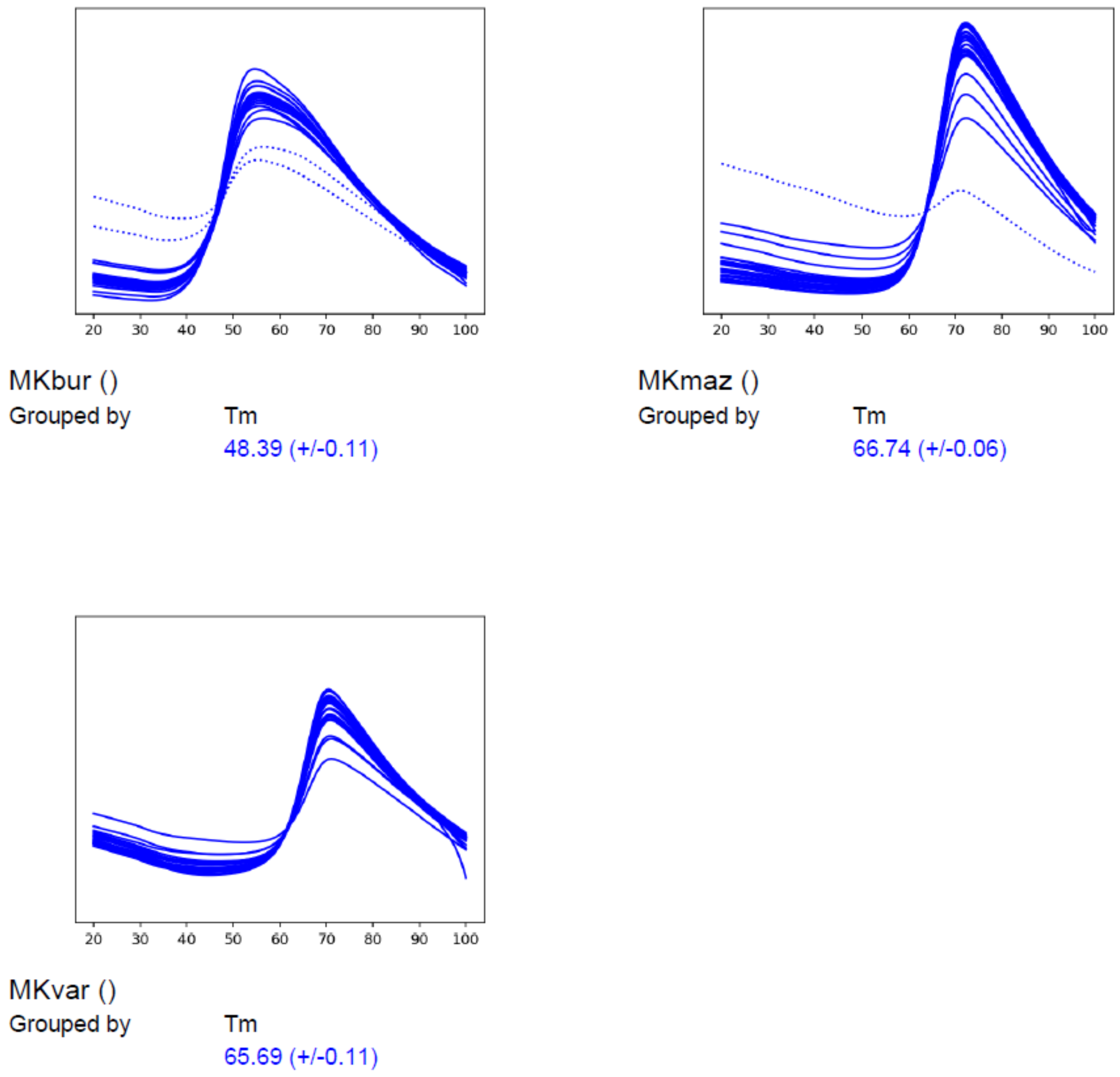

Figure S3 Thermal shift assay curves for MK proteins

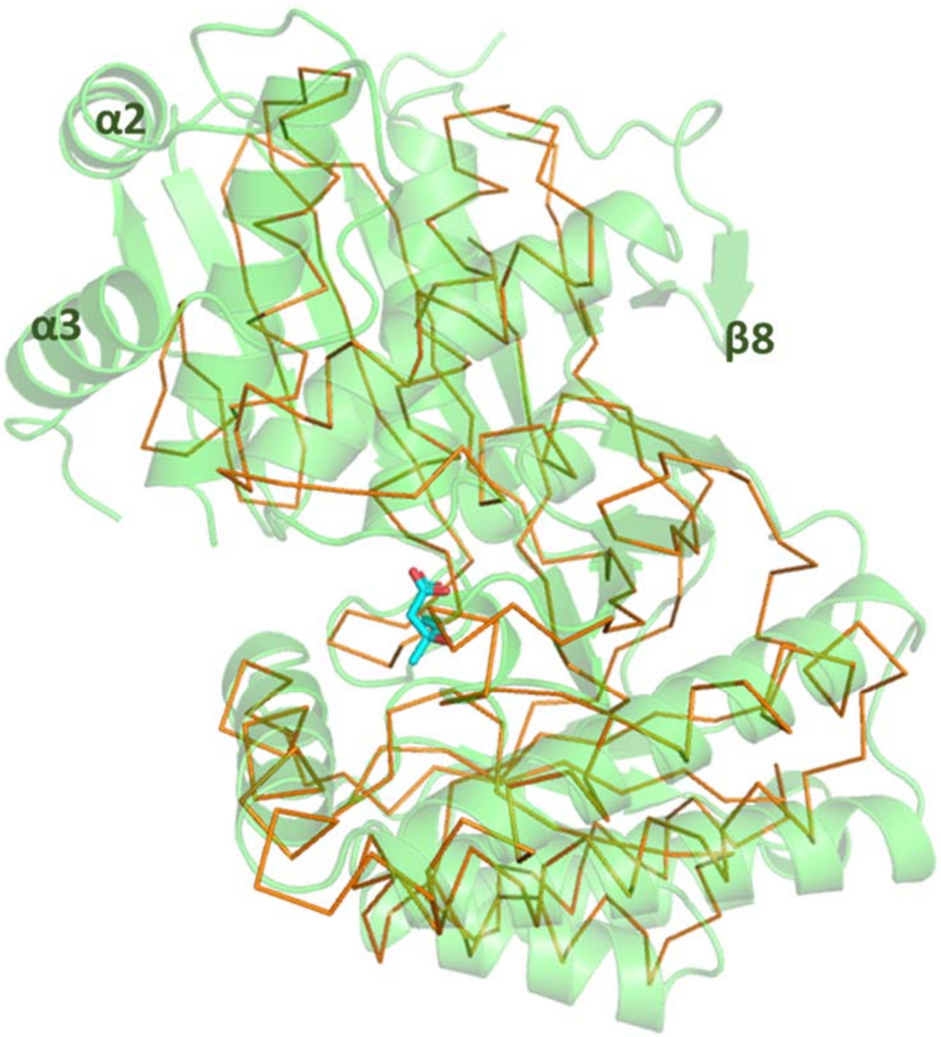

Figure S4 Structural alignment of MKvar (*Ramazzottius varieornatus)* and MKbur (*Methanococcoides burtonii)*, shown in green cartoon and orange ribbon respectively. Helices 2 and 3 and the beta sheet 8 present in MKvar and missing in MKbur are labelled on the figure.

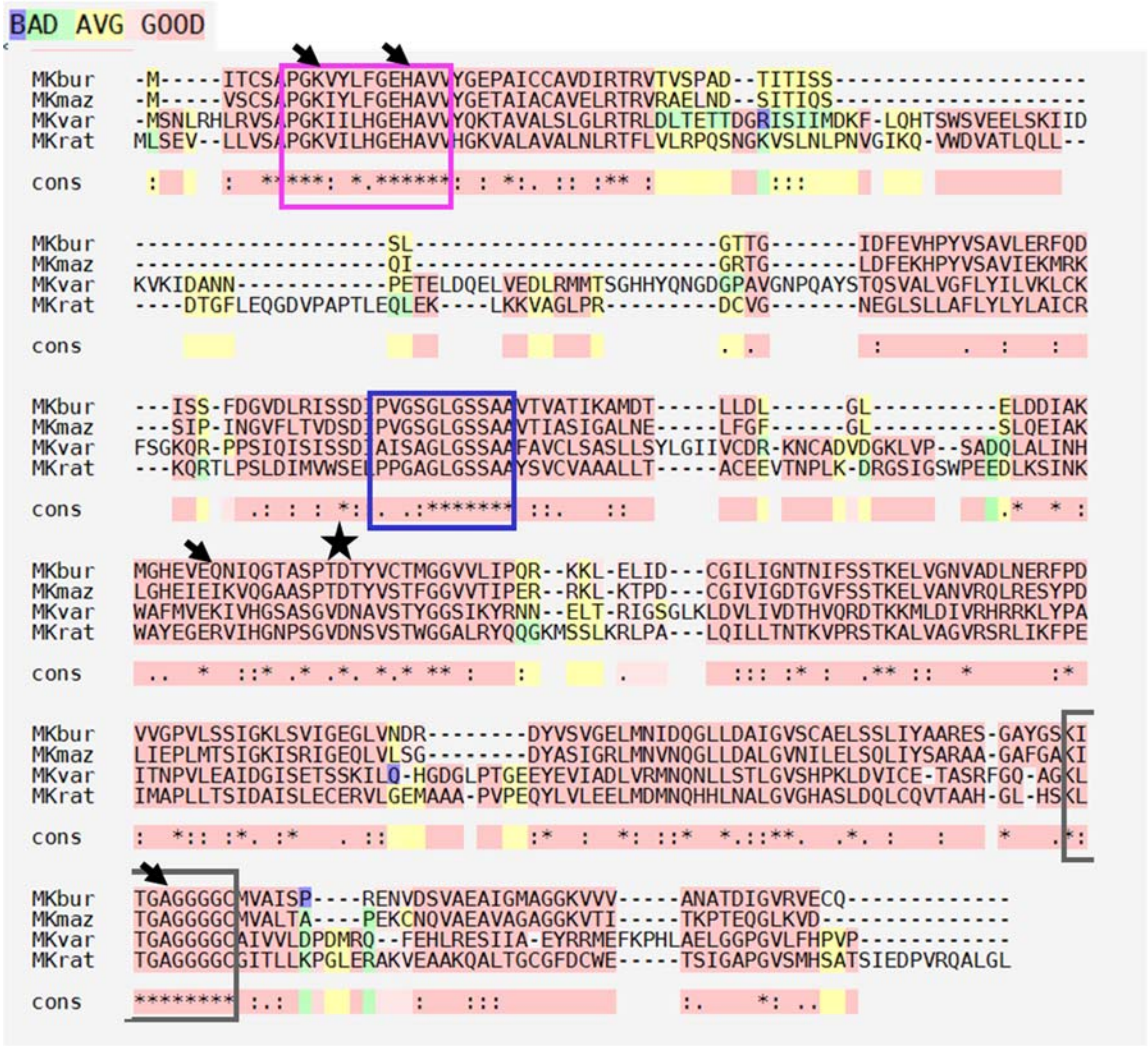

Figure S5 Alignment of the sequences of different mevalonate kinases MKvar (*Ramazzottius varieornatus)*, MKbur (*Methanococcoides burtonii)*, MKmaz (*Methanosarcina mazei*) and MKrat (*Rattus norvegicus*). The colour coding illustration sequence conservation is shown on the top left of the figure. The pink, blue and grey boxes are respectively showing the motif I, II and III of the GHMP superfamily, the black star shows the catalytic amino acids Aspartate and the black arrows conserved amino acids involved in mevalonate binding.

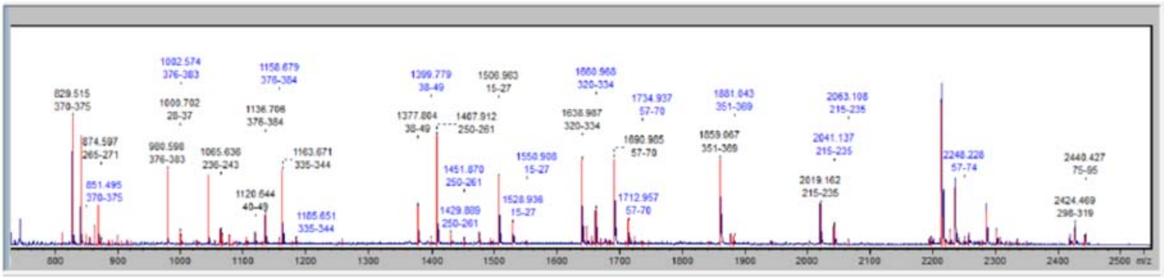


```
Match to: Mevalonate kinase  Score: 41 Expect: 0.00051
MKvar from Ramazzottius varieornatus ((Uniprot Accession A0A1D1VW28)

Fixed modifications: Carbamidomethyl (C)
Variable modifications: Oxidation (M),Cation:Na (DE)
Cleavage by Trypsin: cuts C-term side of KR unless next residue is P
Number of mass values matched: 44
Sequence Coverage: 62%
```

```
  1 MHHHHHHGSM SNLRHLRVSA PGKIILHGEH AVVYQKTAVA LSLGLRTRLD
 51 LTETTDGRIS IIMDKFLQHT SWSVEELSKI IDKVKIDANN PETELDQELV
101 EDLRMMTSGH HYQNGDGPAV GNPQAYSTQS VALVGFLYIL VKLCKFSGKQ
151 RPPSIQISIS SDIAISAGLG SSAAFAVCLS ASLLSYLGII VCDRKNCADV
201 DGKLVPSADQ LALINHWAFM VEKIVHGSAS GVDNAVSTYG GSIKYRNNEL
251 TRIGSGLKLD VLIVDTHVQR DTKKMLDIVR HRRKLYPAIT NPVLEAIDGI
301 SETSSKILQH GDGLPTGEEY EVIADLVRMN QNLLSTLGVS HPKLDVICET
351 ASRFGQAGKL TGAGGGGCAI VVLDPDMRQF EHLRESIIAE YRRMEFKPHL
401 AELGGPGVLF HPVPGS
```

Matched peptides shown in **Bold Red**

Start - End	Observed	Mr(expt)	Mr(calc)	Delta	Miss	Sequence
24 - 36	1506.9630	1505.9557	1505.8354	0.1203	0	K.IILHGEHAVVYQK.T
24 - 36	1528.9360	1527.9287	1527.8174	0.1113	0	K.IILHGEHAVVYQK.T Cation:Na (DE)
37 - 46	1000.7020	999.6947	999.6077	0.0871	0	K.TAVALSLGLR.T
37 - 48	1258.6710	1257.6637	1256.7565	0.9073	1	K.TAVALSLGLRTR.L
47 - 58	1377.8040	1376.7967	1376.6896	0.1071	1	R.TRLDLTETTDGR.I
47 - 58	1399.7790	1398.7717	1398.6715	0.1002	1	R.TRLDLTETTDGR.I Cation:Na (DE)
49 - 58	1120.6440	1119.6367	1119.5408	0.0959	0	R.LDLTETTDGR.I
59 - 65	842.5910	841.5837	840.4391	1.1446	0	R.ISIIMDK.F Cation:Na (DE)
59 - 65	856.6050	855.5977	856.4340	-0.8363	0	R.ISIIMDK.F Oxidation (M); Cation:Na (DE)
66 - 79	1690.9850	1689.9777	1689.8362	0.1415	0	K.FLQHTSWSVEELSK.I
66 - 79	1712.9570	1711.9497	1711.8182	0.1315	0	K.FLQHTSWSVEELSK.I Cation:Na (DE)
66 - 79	1734.9370	1733.9297	1733.8001	0.1296	0	K.FLQHTSWSVEELSK.I 2 Cation:Na (DE)
66 - 83	2225.3180	2224.3107	2225.0721	-0.7614	1	K.FLQHTSWSVEELSKIIDK.V 3 Cation:Na (DE)
84 - 104	2440.4270	2439.4197	2439.2129	0.2068	1	K.VKIDANNPETELDQELVEDLR.M
195 - 203	1027.6370	1026.6297	1027.4369	-0.8072	1	R.KNCADVDGK.L Cation:Na (DE)
196 - 203	901.0400	900.0327	899.3419	0.6908	0	K.NCADVDGK.L Cation:Na (DE)
196 - 203	923.0190	922.0117	921.3239	0.6878	0	K.NCADVDGK.L 2 Cation:Na (DE)
204 - 223	2283.3820	2282.3747	2281.1929	1.1818	0	K.LVPSADQLALINHWAFMVEK.I
204 - 223	2299.3710	2298.3637	2297.1878	1.1759	0	K.LVPSADQLALINHWAFMVEK.I Oxidation (M)
204 - 223	2305.3360	2304.3287	2303.1748	1.1539	0	K.LVPSADQLALINHWAFMVEK.I Cation:Na (DE)
204 - 223	2321.2950	2320.2877	2319.1698	1.1180	0	K.LVPSADQLALINHWAFMVEK.I Oxidation (M); Catio
224 - 244	2019.1620	2018.1547	2018.0069	0.1478	0	K.IVHGSASGVDNAVSTYGGSIK.Y
224 - 244	2041.1370	2040.1297	2039.9888	0.1409	0	K.IVHGSASGVDNAVSTYGGSIK.Y Cation:Na (DE)
245 - 252	1065.6360	1064.6287	1064.5363	0.0925	1	K.YRNNELTR.I
259 - 270	1407.9120	1406.9047	1406.7882	0.1165	0	K.LDVLIVDTHVQR.D
259 - 270	1429.8890	1428.8817	1428.7701	0.1116	0	K.LDVLIVDTHVQR.D Cation:Na (DE)
259 - 270	1451.8700	1450.8627	1450.7521	0.1106	0	K.LDVLIVDTHVQR.D 2 Cation:Na (DE)
274 - 280	874.5970	873.5897	873.5106	0.0791	1	K.KMLDIVR.H
307 - 328	2424.4690	2423.4617	2423.2332	0.2285	0	K.ILQHGDGLPTGEEYEVIADLVR.M
329 - 343	1638.9870	1637.9797	1637.8559	0.1238	0	R.MNQNLLSTLGVSHPK.L
329 - 343	1654.9780	1653.9707	1653.8508	0.1199	0	R.MNQNLLSTLGVSHPK.L Oxidation (M)
344 - 353	1163.6710	1162.6637	1162.5652	0.0985	0	K.LDVICETASR.F
344 - 353	1185.6510	1184.6437	1184.5472	0.0966	0	K.LDVICETASR.F Cation:Na (DE)
360 - 378	1859.0670	1858.0597	1857.9077	0.1520	0	K.LTGAGGGGCAIVVLDPDMR.Q
360 - 378	1875.0760	1874.0687	1873.9026	0.1661	0	K.LTGAGGGGCAIVVLDPDMR.Q Oxidation (M)
360 - 378	1881.0430	1880.0357	1879.8897	0.1460	0	K.LTGAGGGGCAIVVLDPDMR.Q Cation:Na (DE)
379 - 384	829.5150	828.5077	828.4242	0.0835	0	R.QFEHLR.E
379 - 384	851.4950	850.4877	850.4062	0.0815	0	R.QFEHLR.E Cation:Na (DE)
385 - 392	980.5980	979.5907	979.4974	0.0933	0	R.ESIIAEYR.R
385 - 392	1002.5740	1001.5667	1001.4794	0.0874	0	R.ESIIAEYR.R Cation:Na (DE)
385 - 393	1136.7060	1135.6987	1135.5985	0.1002	1	R.ESIIAEYRR.M
385 - 393	1158.6790	1157.6717	1157.5805	0.0912	1	R.ESIIAEYRR.M Cation:Na (DE)
385 - 393	1179.6880	1178.6807	1179.5624	-0.8817	1	R.ESIIAEYRR.M 2 Cation:Na (DE)
394 - 416	2416.4750	2415.4677	2415.2409	0.2268	0	R.MEFKPHLAELGGPGVLFHPVPGS.-

Figure S6 MALDI-TOF spectrum of purified, recombinant MKvar (top) and Mascot peptide mass fingerprint search results for mevalonate kinase from *Ramazzottius varieornatus* (Uniprot Accession A0A1D1VW28) with peptides matching 62% of the target sequence shown in bold.

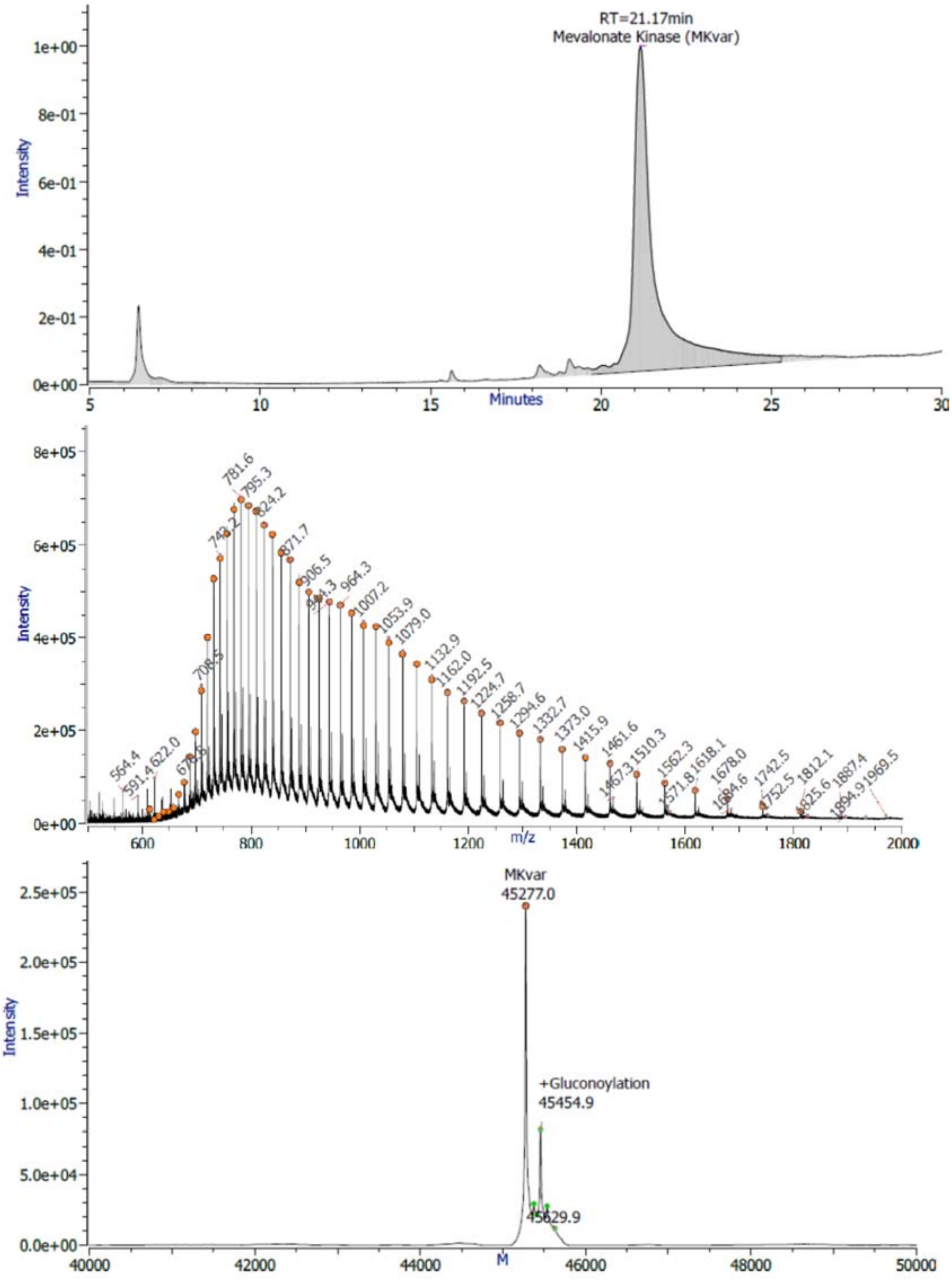

Figure S7 Intact protein analysis of MKvar using LC-MS. The total ion chromatogram shows a sharp peak at 21.17 min (top) containing z = 10 – 70 charge states of purified, recombinant mevalonate kinase in the m/z = 600-2000 Th mass range (middle). The deconvoluted mass spectrum matches the theoretical mass of MKvar (m_{av} = 45,277.4Da) with a minor +178.0Da modification consistent with gluconoylation of the His-tag in *E coli* (bottom).

Table S1 (a) PDBeFold hits for structural resemblance to (a) MKvar's or (b) MKbur's structures, listed by Q-scores.

MK: mevalonate kinase, PMK: phosphomevalonate kinase, NP: non-published.

(a)

organism	Enzyme type (PDB)	*Q score*	%seq identity	RMSD (Å)	Ref.
Burkholderia pseudomallei	heptokinase WcbL (4ut4)	0.49	17	2.0	(Vivoli *et al.*, 2015).
Methanosarcina mazei	MK (6mdf)	0.46	31	2.2	(Miller & Kung, 2018)
Listeria inocua	MK (3k17)	0.45	21	1.9	NP
Streptococcus pneumoniae	PMK (3gon)	0.41	19	2	(Andreassi *et al.*, 2009)
Methanocaldococcus jannaschii	MK (1kkh)	0.41	28	2.4	(Yang *et al.*, 2002)
Bacteroides thetaiotaomicron	Heptokinase (3k85)	0.4	17	2.1	NP
Pyrococcus furiosus	Galactokinase (1s4e)	0.4	20	2.2	(Hartley *et al.*, 2004)
Homo sapiens	MK (2r3v)	0.39	35	2.9	(Fu *et al.*, 2008)

(b)

organism	Enzyme type (PDB)	*Q score*	%seq identity	RMSD (Å)	Ref.
Methanosarcina mazei	MKb (6mde)	0.91	61	0.77	(Miller & Kung, 2018)
Methanocaldococcus jannaschii	MK (1kkh)	0.63	32	1.6	(Yang *et al.*, 2002)
Leishmania major	MK (2hfu)	0.59	26	1.7	(Shafi *et al.*, 2021)
Pyrococcus furiosus	Galactokinase (1s4e)	0.58	24	1.7	(Hartley *et al.*, 2004)

Pyrococcus horikoshii	Galactokinase (2cz9)	0.57	21	1.7	NP
Listeria inocua	MK (3k17)	0.52	23	2.2	NP
Homo sapiens	Galactokinase (6zgx)	0.5	25	1.9	(Mackinnon *et al.*, 2021)
Bacteroides thetaiotaomicron	Heptokinase (3k85)	0.48	17	2.1	NP

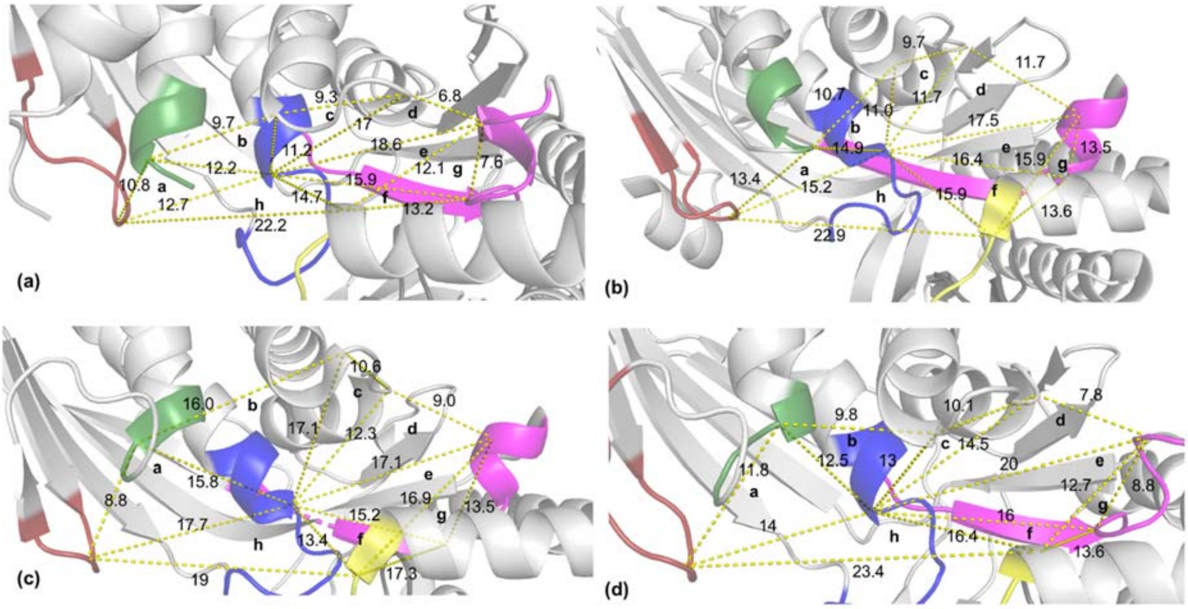

Figure S8 Cartoon representation of (a) MKvar, (b) MKrat, (c) MKmaz, (d) MKbur showing distances between conserved amino acids around the MK's active sites in Å, where the yellow portion is a lid, the blue represents motif II and the dark red a variable nucleotide binding loop, finally the dark green section represents the end of helix α4/α1 (MKvar/MKbur) or equivalent in MKrat and MKmaz. 8 different areas have been labelled a-h and corresponding area calculated are shown in table S2.

Table S2 Surfaces in $Å^2$ of the different areas (a-h) measured in each of the active sites of MKvar, MKbur, MKra and MKmaz as shown in Figure 8.

	MKvar	MKbur	MKrat	MKmaz
a	60.5	69.4	90.3	69.4
b	51.4	57.6	58.8	114.6
c	48.4	63.8	49.6	64.8
d	57.7	46.5	68	53.3
e	59.8	68.4	104.6	98.1
f	90.8	99.9	99.5	98.0
g	45.4	54.5	86.23	106.0
h	88.8	112.9	120.4	113.9
average	62.9	71.6	84.7	89.8
